# Ferroptosis mechanisms and regulations in cardiovascular diseases in the past, present, and future

**DOI:** 10.1007/s10565-024-09853-w

**Published:** 2024-03-21

**Authors:** Wenxi Fang, Saiyang Xie, Wei Deng

**Affiliations:** 1 Department of Cardiology, Renmin Hospital of Wuhan University, Jiefang Road 238, Wuhan, 430060 People’s Republic of China; 2 Hubei Key Laboratory of Metabolic and Chronic Diseases, Wuhan, 430060 People’s Republic of China

**Keywords:** Ferroptosis, Cardiovascular diseases, Treatment, Organelles

## Abstract

Cardiovascular diseases (CVDs) are the main diseases that endanger human health, and their risk factors contribute to high morbidity and a high rate of hospitalization. Cell death is the most important pathophysiology in CVDs. As one of the cell death mechanisms, ferroptosis is a new form of regulated cell death (RCD) that broadly participates in CVDs (such as myocardial infarction, heart transplantation, atherosclerosis, heart failure, ischaemia/reperfusion (I/R) injury, atrial fibrillation, cardiomyopathy (radiation-induced cardiomyopathy, diabetes cardiomyopathy, sepsis-induced cardiac injury, doxorubicin-induced cardiac injury, iron overload cardiomyopathy, and hypertrophic cardiomyopathy), and pulmonary arterial hypertension), involving in iron regulation, metabolic mechanism and lipid peroxidation. This article reviews recent research on the mechanism and regulation of ferroptosis and its relationship with the occurrence and treatment of CVDs, aiming to provide new ideas and treatment targets for the clinical diagnosis and treatment of CVDs by clarifying the latest progress in CVDs research.

**Graphical Abstract:**

• The identification, development history and characterization of ferroptosis.

• The role of different subcellular organelles and organelle-specific regulators in ferroptosis.

• The mechanism of ferroptosis includes iron metabolism, amino acid metabolism, and lipid metabolism.

• The role of ferroptosis in different cardiovascular cells and cardiovascular diseases.

• The treatment efficacy and pathological mechanism involved in ferroptosis and cardiovascular diseases.

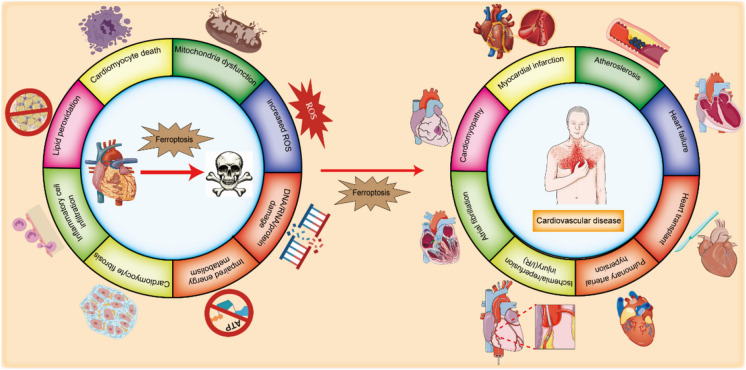

## Introduction

With the ageing of the population, CVDs have become one of the most common causes of death worldwide and seriously threaten physical and mental health. According to the latest data, guidelines suggest that blood lipids, blood pressure, smoking status, diabetes status and obesity are the main risk factors for CVDs (Visseren et al. 2022). Currently, there are various drug and device treatment methods for CVDs, including digitalis, renin angiotensin inhibitors, β receptor blockers, aldosterone receptor antagonists, enkephalin inhibitors, sodium glucose cotransporter 2 inhibitors, diuretics, and cardiac resynchronization therapy (CRT). However, treatment efficacy can be low for quite a few patients with heart failure (HF). Myocardial remodelling is the main pathophysiological process involved in the occurrence and development of HF after various CVDs. In addition to residual myocardial compensatory hypertrophy and myocardial fibrosis, sustained ischaemia and cardiomyocyte death play important roles in the development and exacerbation of cardiac contractile and diastolic dysfunction (McDonagh et al. 2023). According to previous research, cardiovascular pathophysiology includes cell death, inhibition of DNA/RNA/protein synthesis, increased production of reactive oxygen species (ROS), impaired energy metabolism, mitochondrial dysfunction, interstitial fibrosis, autophagy disorders and disorders of intracellular calcium homeostasis (Kong et al. 2022a). Although there are many unknown factors involved in the pathogenesis of CVDs, ferroptosis, which is a type of cell death, has been shown to play an important role in its occurrence and development.

Cell death is a common and conserved characteristic of both physiological and pathological states. Under pathological conditions, different physical, chemical, or mechanical stimuli can cause cardiomyocyte death. For myocardial tissue with limited cell differentiation and regenerative ability, moderate to severe cardiomyocyte death can result in loss of cardiomyocytes, which cannot fully be regenerated and are subsequently replaced by fibrotic scars, seriously affecting cardiac function and leading to HF. Importantly, multiple studies have shown that cardiomyocyte death is associated with various CVDs (McDonagh et al. 2023). In 1972, Kerr creatively used the term "apoptosis" to describe programmed cell death that did not trigger immune activation (Kerr et al. 1972). Later, various forms of cell death, including apoptosis, necroptosis, autophagy, pyroptosis, panotosis, ferroptosis, and cuproptosis, have been discovered (Peng et al. 2022). In 2018, the Nomenclature Committee on Cell Death (NCCD) classified cell death as accidental cell death (ACD) and regulated cell death (RCD) based on functional differences. ACD is an uncontrolled process triggered by lethal stimuli that exceed the cell's ability to adapt and survive (Galluzzi et al. 2018). In contrast, RCD involves a cascade of signalling events carried out by specific effector molecules, including autophagy-dependent cell death, necroptosis, autophagy, pyroptosis, and ferroptosis, among others (Galluzzi et al. 2018).

Ferroptosis is a type of cell death that involves the Fenton reaction, lipid peroxidation and ROS accumulation (Stockwell 2022). At present, the regulatory pathways known to be involved in ferroptosis include iron metabolism, glutathione metabolism, and lipid metabolism. Recently, many studies have shown that ferroptosis is involved in various CVDs (Fang et al. 2023). Therefore, understanding the pathogenesis of CVDs this will help us identify potential therapeutic targets for CVDs and improve CVDs treatment.

## Identification and characterization of ferroptosis

**Fig. 1:**
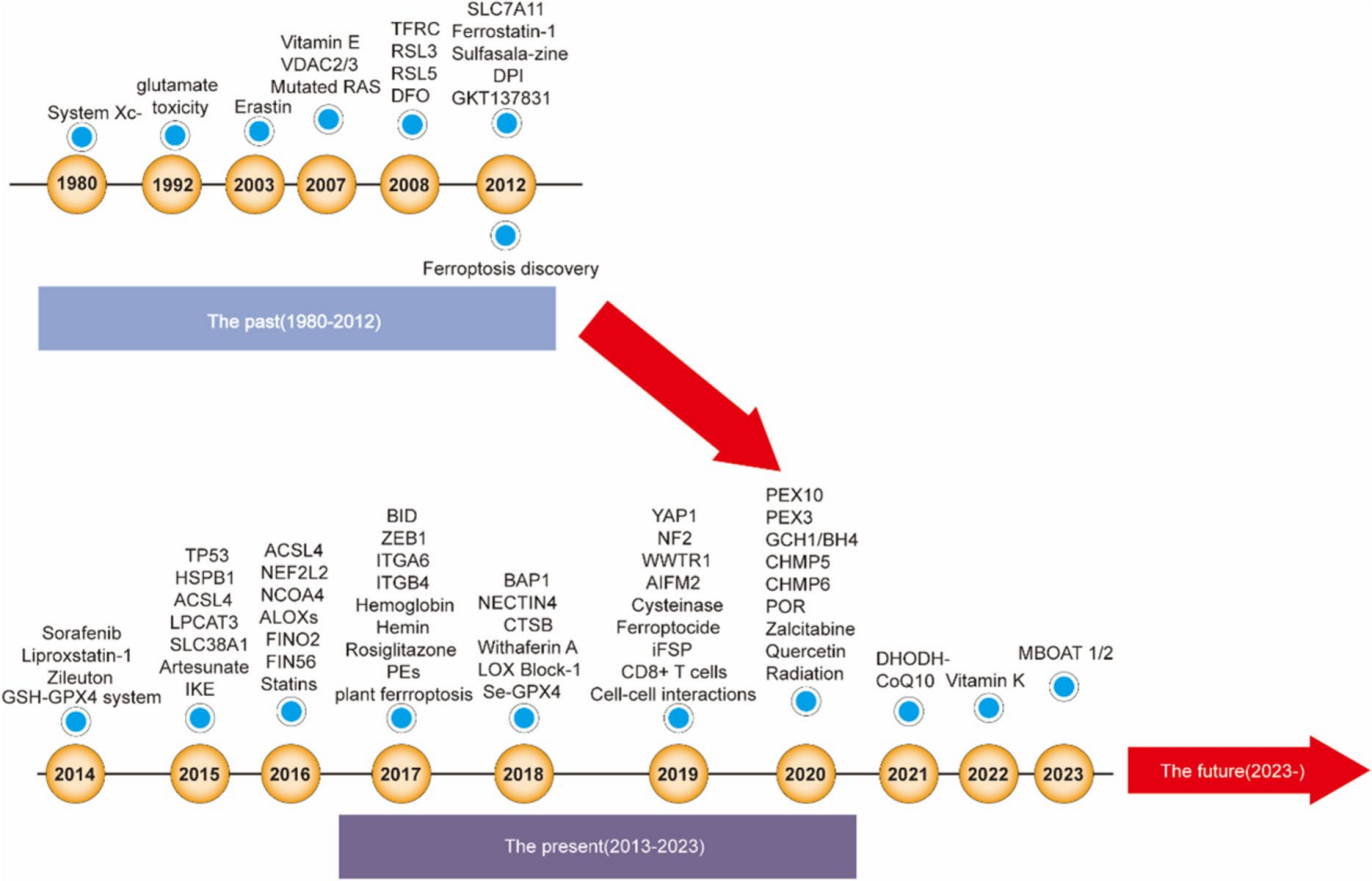
Timeline of the identification and characterization of ferroptosis (past, present, future). TFRC: Transferrin receptor, Se-GPX4: Se-Glutathione peroxidase 4, RSL3: RAS-selective lethal 3, RSL5: RAS-selective lethal 5, DFO: Deferoxamine, SLC7A11: Solute carrier family 7 member 11, DPI: Diphenyliodonium chloride, GPX4: Glutathione peroxidase 4, GSH: Glutathione, TP53: Tumour protein p53, HSPB1: Heat shock protein beta-1, ACSL4: Acyl-CoA synthetase long-chain family member 4, LPCAT3: Lyso-phosphatidylcholine acyltransferase-3, SLC38A1: Solute carrier family 38 member 1, IKE: Imidazole ketone erastin, NEF2L2: Nuclear factor erythroid 2-like 2, NCOA4: Nuclear receptor coactivator 4, LOX: Lipoxygenase, FINO2: 1,2-dioxolane, FIN56: Ferroptosis inducing 56, BID: BH3 interacting domain death agonist, ZEB1: Zinc finger E-box-binding homeobox protein 1, ITGA6: Integrin alpha-6, ITGB4: Integrin beta 4, PEs: Phosphatidylethanolamines, BAP1: BRCA1-associated protein 1, NECTIN4: Nectin cell adhesion molecule-4, CTSB: cathepsin B, YAP1: Yes-associated protein 1, NF2: Neurofibromin, CoQ_10_: Coenzyme Q10, WWTR1: Transcriptional coactivator with PDZ-binding motif, AIFM2: Apoptosis-inducing factor mitochondria-associated 2, IFSP1: Ferroptosis-suppressor protein 1 inhibitor, PEX10: Peroxisome biogenesis factor 10, PEX3: Peroxisome biogenesis factor 3, DHODH: Dihydroorotate dehydrogenase (quinone), GCH1: GTP cyclohydrolase 1, BH4: Tetrahydrobiopterin, CHMP5: Charged multivesicular body protein 5, CHMP6: Charged multivesicular body protein 6, POR: NADPH-cytochrome P450 reductase, MBOAT 1/2: Membrane-bound O-acyltransferase domain-containing 1 and 2

Ahat erastin could kill cancer cells by inducing non-apoptotic regulatory death in RAS-expressing cells. This drug induces ferroptosis, which preceded the discovery of ferroptosis (Dolma et al. 2003). Similarly, Nicholas Yagoda reported that when an iron chelating agent was used, cell death could be inhibited, with the level of ROS increasing (Yagoda et al. 2007). In addition, Scott J Dixon et al. reported that erastin and ferrostatin-1(Fer-1) could exacerbate and inhibit ferroptosis, respectively. This concept was proposed to constitute a lethal pathway with clear characteristics that differ from those of apoptosis, necrosis and other regulatory cell death types, and this concept was used to selectively destroy RAS-mutant tumour cells or protect nerve cells exposed to specific oxidative conditions (Dixon et al. 2012) (Fig. 1).

Ferroptosis is a kind of regulatory cell death caused by the accumulation of ROS due to abnormal iron metabolism, lipid peroxidation, and amino acid metabolism. The differences between ferroptosis and other cell death modes can be illustrated based on these characteristics (Table 1).

**Table 1:** Differences between ferroptosis and other types of cell death

Cell death	Classification	Morphological characteristics	Molecular biology	Phenotypic genes	Regulators	Trigger inflammation
Ferroptosis	RCD	Swollen mitochondria, increased mitochondrial membrane density, smaller mitochondria, decreased or disappeared mitochondrial crista, increased lamellar phenotype, and increased autophagy	Depletion of GSH, decline in GPX4 activity, increase in lipid peroxidation products and accumulation of ROS	GPX4, PTGS2, FSP1, NRF2, NCOA4	Erastin, Ferrostatins Liproxstatins, Sorafenib, Deferoxamine	Yes
Apoptosis	RCD	Cellular shrinkage, chromatin condensation, nuclear fragmentation, formation of apoptotic bodies	Ca^2+^/mg^2+^-dependent endonuclease and calpain activation, DNA fragmentation, increased activity of cysteine aspartate protease and apoptosis protease activity	Caspase, Bcl-2, Bax, P53	Staurosporine, Z-VADFMK, Hypoxia, NAIP	No
Pyroptosis	RCD	Scorched corpuscles, broken plasma membrane cells, forming pores, and swollen and expanded cells	Formation of inflammatory bodies, activation of caspase and gasfermin, and release of many pro-inflammatory factors	Caspase-1, NLRP3, GSDMD,Caspase-3, GSDME	Ivermectin, Necrosulfonamide	Yes
Necroptosis	RCD	Cytoplasm swelling, organelle enlargement, chromatin noncoagulation, cell membrane rupture, cell content leakage, lysosome rupture, nuclear chromatin dissolution	ROS generation, DNA degradation, pro-inflammatory factor formation, necrosis body formation	CAMKII, RIPK3, RIPK1, MLKL, FADD, mPTP	Necrostatin-1, KN-93,TNF-α	Yes
Autophagy	-	Accumulation of double-membraned autolysosomes	AMPK, mTOR, ATG5, ATG7, Beclin 1	ATGs, LC3, P62, Pink1, Parkin2	3-MA, Wortmannin, LY294002	No
Cuproptosis	RCD	Mitochondrial shrinkage, mitochondrial membrane rupture	Copper accumulation, Protein lipoylation	FDX1, LIAS, LIPT1, DLAT, GLS, MTF	TTM	Yes

*RCD* regulated cell death, *GSH* glutathione, *GPX4* glutathione peroxidase 4, *PTGS2* prostaglandin-endoperoxide synthase 2, *FSP1* ferroptosis-suppressor protein 1, *NRF2* nuclear factor erythroid 2-Related factor 2, *NCOA4* nuclear receptor coactivator 4, *Bcl-2* b-cell lymphoma-2, *Bax* bCL-2 associated protein X, *NAIP* neuronal apoptosis inhibitory protein, *NLRP3* nod-like receptor thermal protein domain associated protein 3, *CAMKII* calcium/calmodulin-dependent protein kinase II, *RIPK3* recombinant receptor-interacting serine threonine kinase 3, *RIPK1* recombinant receptor-interacting serine threonine kinase 1, *MLKL* mixed lineage kinase domain-like, *FADD* fas-associated death domain, *MPTP* 1-methyl-4-phenyl-1.2.6-tetrahydropyridine, *TNF-α* tumour necrosis factor-α, *ROS* reactive oxygen species, *ATG5* autophagy-related gene 5, *ATG7* Autophagy-related gene 7, *AMPK* amp-activated protein kinase, *mTOR* the mechanistic target of rapamycin, *FDX1* ferredoxin 1, *LIAS* lipoic acid synthetase, *LIPT1* lipoyltransferase 1, *DLAT* dihydrolipoamide S-acetyltransferase, *GLS* glutaminase, *MTF* metal regulatory transcription factor, *TTM* tetrathiomolybdate, *ATGs* autophagy-related genes, *LC3* microtubule-associated protein light chain 3, *P62* Sequestosome 1, *Pink1* pten-induced putative kinase 1, *Parkin2* e3 ubiquitin ligase 2, *3-MA* 3-methyladenine, *GSDMD* gasdermin D, *GSDME* gasdermin E, *m-PTP* mitochondrial permeability transition pore

## Subcellular organelles drive ferroptosis (Fig. 2)

### Plasma membrane rupture

The end stage of ferroptosis involves permeabilization of the plasma membrane. The endosomal sorting complex required for transport (ESCRT III) complex can repair plasma membrane damage and alleviate ferroptosis (Pedrera et al. 2021). In addition, ferroptosis signals spread to adjacent cells, possibly through release of oxidized lipids through extracellular vesicles, leading to ferroptosis in adjacent cells (Riegman et al. 2020). Finally, Ferroptosis-suppressor protein 1 (FSP1) can exert its defensive function against ferroptosis by reducing the amount of Coenzyme Q10 (CoQ_10_) located at the plasma membrane (Bersuker et al. 2019).

**Fig. 2:**
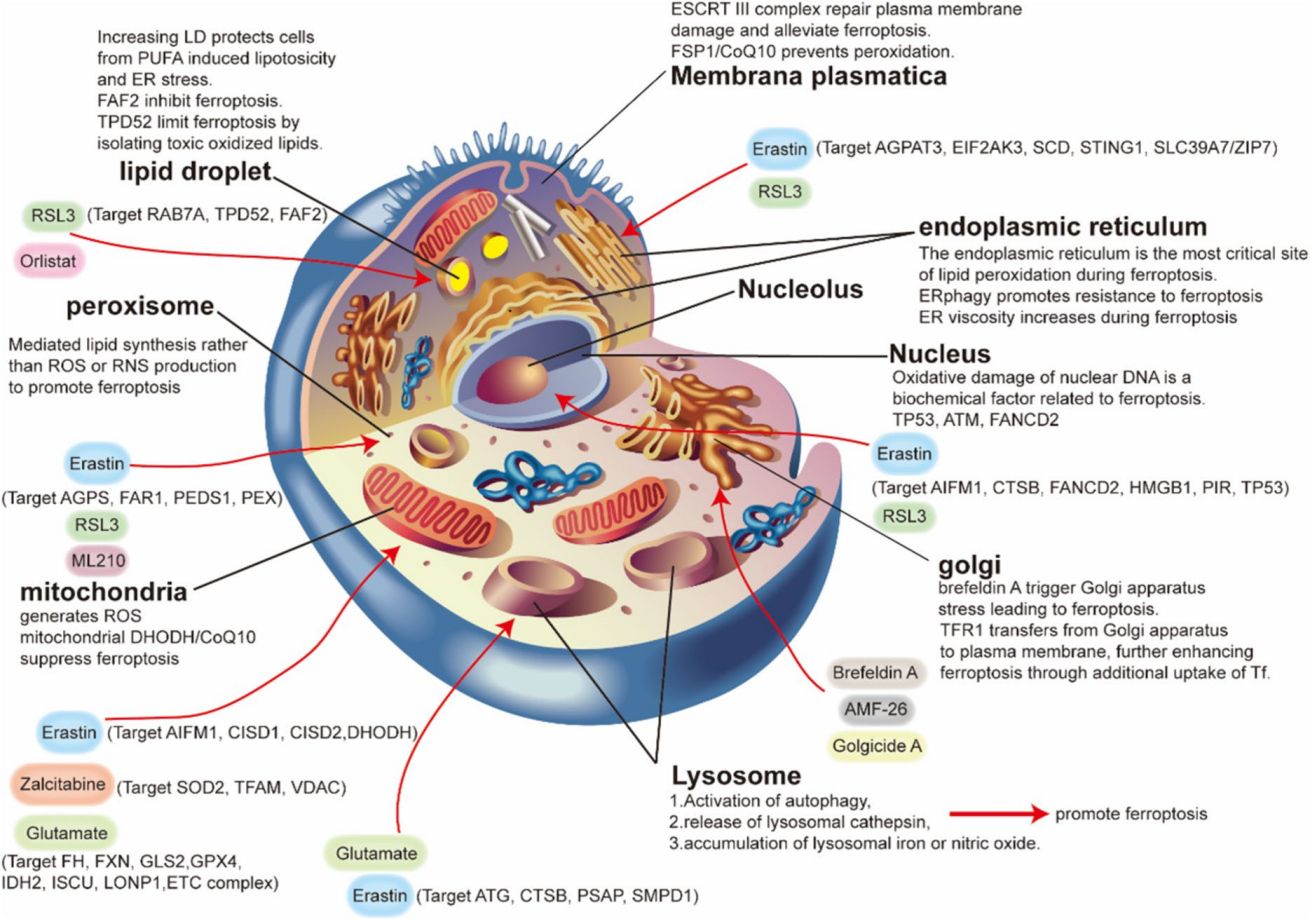
Role of different subcellular organelles and organelle-specific regulators in ferroptosis. AGPAT3: 1-Acylglycerol-3-phosphate O-acyltransferase 3, AGPS: Alkylglycerone phosphate synthase, AIFM1/AIF: Apoptosis-inducing factor mitochondria-associated 1, AMF-26: 2-methylcoprophilinamide, ATG: Autophagy-related gene, ATM: ATM serine/threonine kinase, CISD1: CDGSH iron sulfur domain 1, CISD2: CDGSH iron sulfur domain 2, CTSB: cathepsin B, DHODH: Dihydroorotate dehydrogenase (quinone), EIF2AK3/PERK: Eukaryotic translation initiation factor 2 alpha kinase 3, ESCRT III: The endosomal sorting complexes required for transport, ER: Endoplasmic reticulum, ETC: Electron transport chain, FAF2: Fas-associated factor family member 2, FANCD2: FA complementation group D2, FAR1: Fatty acyl-CoA reductase 1, FH: Fumarate hydratase, FSP1: Ferroptosis-suppressor protein 1, FXN: Frataxin, GLS2: Glutaminase 2, GPX4: Glutathione peroxidase 4, HMGB1: High-mobility group box 1, IDH2: Isocitrate dehydrogenase (NADP[ +]) 2, ISCU: Iron-sulfur cluster assembly enzyme, LDs: Lipid droplets, LONP1: Lon peptidase 1, PEDS1/TMEM189: Plasmanylethanolamine desaturase 1, PEX: Peroxisomal biogenesis factor, PIR: Pirin, PUFA: Polyunsaturated fatty acid, PSAP: Prosaposin, RAB7A: Member RAS oncogene family, RNS: Reactive nitrogen species, ROS: Reactive oxygen species, RSL3: RAS-selective lethal 3, SCD/SCD1: Stearoyl-CoA desaturase, SLC39A7/ZIP7: Solute carrier family 39 member 7, SMPD1/ASM: Sphingomyelin phosphodiesterase 1, SOD2: Superoxide dismutase 2, STING1/TMEM173: Stimulator of interferon response cGAMP interactor 1, TFAM: Transcription factor A, mitochondrial, TFR1: Transferrin Receptor 1, TP53: Tumour protein p53, TPD52: Tumour protein D52, VDAC: Voltage-dependent anion channel

### Endoplasmic reticulum

The endoplasmic reticulum (ER) is an organelle responsible for protein synthesis and processing as well as lipid secretion. The ER is the source of most cell membrane lipids in other organelles, and the ER is spatially and functionally connected to other organelles, such as mitochondria and lysosomal systems (Phillips and Voeltz 2016). ER stress (ERS) triggers an unfolded protein response to restore protein homeostasis, but when cells cannot restore homeostasis, ERS can lead to cell death (Hetz et al. 2020). ERS plays dual roles in ferroptosis. For example, erastin can specifically inhibit cysteine uptake through system Xc^−^, leading to ERS. Activating transcription factor 4 (ATF4) participates in heat shock protein family A member 5 (HSPA5) expression and upregulation of solute carrier family 7 member 11 (SLC7A11) to inhibit ferroptosis, thus enhancing the resistance of pancreatic cancer cells or glioma cells to ferroptosis caused by gemcitabine or dihydroartemisinin (Dixon et al. 2014). On the other hand, transcription of the glutathione (GSH)-degrading enzyme chac glutathione specific gamma-glutamylcyclotransferase 1 (CHAC1) mediated by ATF4 enhances the ferroptosis of breast cancer cells induced by artesunate or cystine starvation (Chen et al. 2017). Furthermore, fer-1 may exert its anti-ferroptotic effect through its accumulation in the ER rather than through its accumulation in lysosomes and mitochondria (Gaschler et al. 2018). The viscosity of the ER increases during ferroptosis, possibly due to aggregation of polyunsaturated fatty acids (PUFAs) and phospholipids, leading to hardening of the ER (Liu et al. 2021a).

In addition to ERS, other pathways affect ferroptosis. For example, zinc transporter solute carrier family 39, member 7 (ZIP7) controls the transport of zinc from the ER to the cytoplasm, and ZIP7 knockdown may protect against ferroptosis by upregulating homocysteine-responsive endoplasmic reticulum-resident ubiquitin-like domain member 1 (HERPUD1), a well-known gene induced during ERS. This process may be related to involvement of zinc in ER and nuclear communication (Chen et al. 2021a). Exogenous mono-unsaturated fatty acids (MUFA) treatment can reduce the sensitivity of plasma membrane lipids to oxidation within a few hours. This effect requires activation of MUFAs by acyl-CoA synthetase long-chain 3 (ACSL3) to resist ferroptosis and is not related to formation of lipid droplets (Magtanong et al. 2019).

### Mitochondria

Mitochondria are involved in energy metabolism, signal transduction, and regulation of death pathways. They are also the main source of ROS and the main site for iron ions to exert their effects in cells (Gao et al. 2019). When ferroptosis occurs, mitochondrial morphology changes, including mitochondrial shrinkage, ridge enlargement, and outer membrane rupture. Abnormal mitochondrial dynamics and dysfunction increase susceptibility to ferroptosis, and mitochondria act as a double-edged sword in ferroptosis (Fang et al. 2023; Dixon et al. 2012; Friedmann Angeli et al. 2014). On one hand, mitochondria can promote ferroptosis. Because ferroptosis is the main source of ROS, accumulation of a large amount of ROS makes cells prone to ferroptosis (Fang et al. 2023; Ali et al. 2019; Jan et al. 2017). In addition, mitochondrial glutamine breakdown can promote ferroptosis caused by amino acid starvation (Gao et al. 2019). Mitochondria play a central role in cellular iron metabolism, and high concentrations of iron make them ideal sites for inducing ferroptosis. Early studies have shown that cancer cell lines with mitochondrial DNA deficiency are equally sensitive to ferroptosis as are wild-type mitochondrial DNA cells (Dixon et al. 2012). Cells that undergo mitochondrial autophagy and consume mitochondria can still undergo ferroptosis.

On the other hand, mitochondria can resist ferroptosis. Fatty acid β-oxidation occurs mainly in mitochondria, and β-oxidation inhibits lipid peroxidation by reducing accumulation of PUFAs. Upon inactivation of Glutathione peroxidase 4 (GPX4), mitochondrial Dihydroorotate dehydrogenase (DHODH) can protect cells from ferroptosis by inhibiting lipid peroxidation (Mao et al. 2021). Some proteins, such as cysteine desulfurase (NFS1) and frataxin (FXN), participate in synthesis of Fe-S clusters and have anti-ferroptotic effects. Mitochondrial DNA deficiency and subsequent mitochondrial dysfunction can increase the sensitivity of liver cells to ferroptosis in patients with mitochondrial DNA deficiency under iron overload conditions (Guo et al. 2021). Mitochondrial DNA stress also triggers autophagy-dependent ferroptosis by activating the cyclic GMP-AMP synthase-stimulator of interferon genes (cGAS-STING) pathway (Hopfner and Hornung 2020).

### mitochondria-associated membranes

Mitochondria-associated membranes (MAMs) is a contact site between ER and mitochondria (Zhang et al. 2024). MAMs contains many proteins with diverse functions, regulating various cellular biological functions between the ER and mitochondria, such as lipid metabolism, calcium signaling, inflammatory immunity, ERS, mitochondrial quality control, and cell death (Janikiewicz et al. 2018). The composition of MAMs includes: 1) Ca^2+^ channels located on the ER or outer mitochondrial membrane, such as inositol 1,4,5-triphosphate receptor (IP3R) and voltage dependent anion channel 1 (VDAC l) (Furuichi et al. 1989; Szabadkai et al. 2006), 2) lipid synthesis and transferases (Voelker 2005), 3) various molecular chaperones, such as glucose regulated protein 75 (GRP75) and sigma 1 receptor (S1R) (Szabadkai et al. 2006; Hayashi and Su 2007), 4) Enzymes involved in ER redox reactions, such as endoplasmic reticulum oxidoreductases α1 (Erolα) (Pollard et al. 1998; Anelli et al. 2012), 5) Mitochondrial Rho GTPases 1 (Miro1) and Mitofusins 2 (MFN2), which are involved in mitochondrial activity (Fransson et al. 2003; Saotome et al. 2008; Kornmann et al. 2011). The role of calcium in ferroptosis is of great interest, although it remains controversial (Dixon et al. 2012; Pedrera et al. 2021; Zhang et al. 2024; Gleitze et al. 2021; Murphy et al. 1988; Davis and Maher 1994; Xin et al. 2022). MAMs are involved in the trafficking of phosphatidylserine into mitochondria and phosphatidylethanolamine out of mitochondria, acting as a mechanical link between ER and mitochondria (Flis and Daum 2013). MAMs modulate ferroptosis through controlling Ca^2+^ and lipid transfer from ER to mitochondria. Calcium transfer–mitochondrial ROS axis and lipid transfer–PUFA-containing triacylglycerols (TAG) accumulation axis controlled by MAMs have an important role in ferroptosis execution (Zhang et al. 2024). A variety of enzymes enriched in MAMs including Ero1α and ER resident protein 44 (ERp44) can lead to excessive production of mtROS. Ero1α induces IP3R oxidation, resulting in the dissociation of ERp44 from IP3R, thereby enhancing the transfer of Ca^2+^ from ER to mitochondria, resulting in excessive production of mtROS (Liu and Yang 2022). DHODH, along with mitochondrial targeted GPX4, has been found to be a goalkeeper for mitochondrial lipid peroxidation (Mao et al. 2021). CGI1746 therapy or chemical and genetic inhibition of MAMs related genes by acting on sigma-1 receptors (σ1R) located in the MAMs, which leads to impaired Ca^2+^ and lipid transport from the ER to mitochondria, significantly blocking ferroptosis and lipid peroxidation (Zhang et al. 2024). p66Shc, a member of Shc protein family, has been confirmed presence in MAMs, which plays a role in signal transduction and cell response to oxidative stress. It is reported that p66Shc promotes mtROS production by phosphorylation at Ser36. Notably, p66Shc Ser36 phosphorylation also initiates the translocation of p66Shc to MAMs, where it could participate in mtROS production (Lebiedzinska et al. 2009; Huang et al. 2021).

### Peroxisome

Peroxisomes are organelles that produce ROS and reactive nitrogen species (RNS) through prooxidants such as xanthine dehydrogenase (XDH) and nitric oxide synthase 2 (NOS2) (Smith and Aitchison 2013). In contrast, peroxisomes contain antioxidant enzymes such as catalase (CAT), superoxide dismutase 1 (SOD1), peroxiredox protein 5 (PRDX5), and glutathione S-transferase kappa 1 (GSTK1) (Fransen et al. 2012). However, a recent study screened for genes involved in peroxisome function through clustered regularly interspaced short palindromic repeats (CRISPR)-mediated promotion of ferroptosis via production of peroxisomal ether lipids, such as plasma proteins, rather than ROS or RNS, which are considered driving factors of ferroptosis and require involvement of peroxisome Fe (II) in this process (Zou et al. 2020). Neurons from plasmalogen-deficient (PEX7 knockout) mice are more susceptible to ROS-mediated damage than are those from other mice (Luoma et al. 2015), indicating that ether phospholipids might also act as endogenous antioxidants. In addition to lipid synthesis and redox balance, peroxisomes are involved in biosynthesis and signalling of steroid and peptide hormones, which in turn might indirectly impinge on the regulation of ferroptosis (Weinhofer et al. 2013).

### Lysosome

**Fig. 3:**
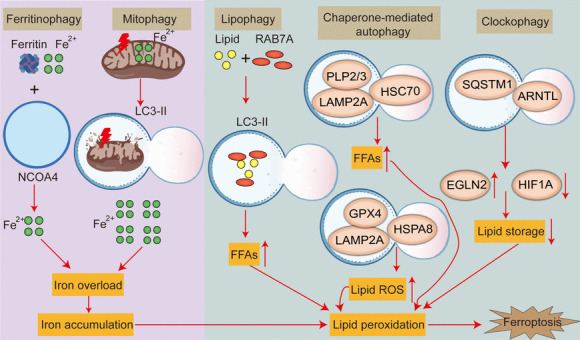
The role of autophagy in ferroptosis. 5 types of autophagy including ferritinophagy, mitophagy, lipophagy, chaperone-mediated autophagy and clockphagy. NCOA4: nuclear receptor coactivator 4, LC3-II: light chain 3-II, HSC70: heat shock cognate 71 kDa protein, HSPA8: heat shock 70 kDa protein 8, LAMP2A: lysosome-associated membrane protein 2A, ARNTL: Aryl hydrocarbon receptor nuclear translocator-like protein 1, SQSTM1: sequestosome-1, GPX4: glutathione peroxidase 4, RAB7A: Member RAS Oncogene Family, HIF1A: Hypoxia-inducible factor 1 alpha, FFAs: free fatty acids, EGLN2: Egl-9 family hypoxia-inducible factor 2

Lysosomes can degrade and circulate essential nutrients in the body, participating in ferroptosis through three main mechanisms: 1) activation of autophagy, 2) release of lysosomal cathepsin, and 3) accumulation of lysosomal iron or nitric oxide. Autophagy is a lysosomal-dependent degradation pathway that is primarily performed by sequential contribution of autophagy-associated (ATG) proteins (Hou et al. 2016). Several selective autophagy pathways promote ferroptosis by bypassing different pathways. At present, there are five pathways that link ferroptosis and autophagy, including ferritinophagy, clock autophagy, molecular chaperone-mediated autophagy, mitophagy and lipophagy, which involves degradation of lipid droplets (LDs) in cells through autophagy (Fig. 3). Free fatty acids (FFAs) produced by lipid autophagy pass through mitochondrial β-oxidation to promote ATP production. Lipid autophagy promotes RAS-selective lethal 3 (RSL3)-induced lipid peroxidation and ferroptosis, and tumor protein D52 (TPD52) overexpression promotes lipid storage or inhibits lipid-related autophagy to effectively inhibit RSL3-induced lipid peroxidation and ferroptosis. Clock autophagy is a selective autophagy process. Aryl hydrocarbon receptor nuclear translocator-like protein 1 (ARNTL1), which is the core protein of the biological clock, can be degraded through p62-mediated selective autophagy, leading to an increase in expression of Prolyl Hydroxylase Domain 1 (PHD1), promoting lipid peroxidation in cells and further promoting ferroptosis. Chaperone-mediated autophagy (CMA) requires a molecular chaperone to recognize the lysine, phenylalanine, glutamic acid, arginine, glutamine (KFERQ) amino acid sequence in the substrate and then bind with lysosome-associated membrane protein 2A (LAMP2A) to enter the lysosome, after which the substrate is degraded accordingly. GPX4 can eliminate lipid peroxides and protect cells from ferroptosis. GPX4 can interact with Heat shock 70 kDa protein 8 (HSPA8), which is a molecular chaperone, and can then be degraded by CMA. Overexpression of LAMP2A can promote CMA-mediated degradation of GPX4, leading to ferroptosis. However, in contrast to HSPA8, heat shock protein family A member 5 (HSPA5) inhibits degradation of GPX4 by interacting with GPX4, thereby inhibiting ferroptosis (Li et al. 2021a). Mitophagy also plays an important role in ferroptosis (Xie et al. 2023). Classic mitophagy pathways include PTEN-induced kinase 1 (PINK1)/ parkin RBR E3 ubiquitin protein ligase (Parkin), Bcl-2 19-kDa interacting protein 3 (BNIP3)/ NIP3-like protein X (Nix) and FUN14 domain containing 1 (FUNDC1) pathway (Ajoolabady et al. 2022). In the early stages of iron overload, a large amount of released iron is transported as a buffer to mitochondria, and mitochondrial autophagy may isolate iron into mitochondrial autophagosomes, reducing the source of ROS for ferroptosis. However, excessive iron overload leads to mitochondrial damage, induces further mitochondrial autophagy, and provides an additional source of iron for lipid peroxidation. Ultimately, extensive mitochondrial autophagy releases iron, ROS, and peroxidized lipids from mitochondria at toxic levels, thereby activating various ROS induced ferroptosis (Lee et al. 2023). BAY 87-2243 inhibits mitochondrial respiratory chain complex I, leading to 1-methyl-4-phenyl-1-1,2,3,6-tetrahydropyridine (mPTP) opening and decreased mitochondrial membrane potential, which in turn increases ROS activation of ferroptosis by stimulating mitochondrial autophagy, while knocking down PINK1 inhibits BAY induced ferroptosis (Basit et al. 2017). Zalcitabine induces oxidative mtDNA damage and decreased mitochondrial function as well as degradation of the mitochondrial (Lon protease 1)(LONP1)-dependent mitochondrial transcription factor A (TFAM). These effects result in the activation of the DNA damage sensing CGAS-STING1 pathway, inducing autophagy and subsequently causing autophagy-dependent ferroptosis (Li et al. 2021b). BAY 11-7085 (inhibitor of NK-κB activation) induced ferroptosis via nuclear factor-E2-related factor 2- (Nrf2-) SLC7A11—heme oxygenase-1 (HO-1) pathway and causes compartmentalization of HO-1 into the nucleus and mitochondria, and followed mitochondrial dysfunctions, leading to lysosome targeting for mitophagy (Chang et al. 2018).

Release of lysosomal cathepsin B (CTSB), especially, is currently considered one of the causes of ferroptosis. CTSB-mediated DNA damage can activate STING1-dependent autophagy (Kuang et al. 2020). Cleavage of histone H3 may alter expression of iron phagocytic-related genes (Nagakannan et al. 2021). In addition, activation of nuclear transcription factor EB (TFEB) can inhibit lysosomal-dependent ferroptosis by inducing expression of the antioxidant superoxide dismutase 1 (SOD1) gene (Li et al. 2019a). Involvement of the ER in relocating 1,2-dioxolane (FINO2)-type peroxides to lysosomes can lead to ferroptosis (Liu et al. 2020). Moreover, knocking out the lysosomal protein proapsin causes lipofuscin accumulation, which drives accumulation of lysosomal iron and ROS, followed by ferroptosis in neurons (Tian et al. 2021).

### Golgi apparatus

The Golgi apparatus processes, modifies, categorizes, and packages proteins synthesized by the ER and ultimately moves to specific parts of the cell or secretes them out of the cell (Alborzinia et al. 2018**).** Golgi inducers (AMF-26/M-CO-PA, brefeldin A, GCA) promote ferroptosis in HeLa cells, which can be prevented by overexpressing SLC7A11 or GPX4 or by knocking out ACSL4. In addition, drugs such as brefeldin A trigger Golgi stress and lead to ferroptosis. Ferroptosis inhibitors can reverse the inhibitory effect of brefeldin A on protein secretion in the Golgi apparatus, protecting the morphology and function of the Golgi apparatus. Notably, a sublethal dose of the ferroptosis inducer erastin can inhibit, rather than promote, the lipid peroxidation induced by brefeldin A, which involves the sulfur transfer pathway and partially restores the balance of the Golgi apparatus (Alborzinia et al. 2018). Transferrin Receptor 1 (TFR1) is translocated from the Golgi apparatus to the plasma membrane, where it enhances ferroptosis through additional uptake of transferrin (TF) (Feng et al. 2020).

### Lipid droplets

LDs are storage organelles at the centre of lipid and energy homeostasis. LDs also dynamically contact other organelles (such as mitochondria, the ER, peroxisomes, and lysosomes) to promote the exchange of lipids, metabolites, and ions (Olzmann and Carvalho 2019). The balance between the degradation and storage of LDs can affect their sensitivity to ferroptosis. For example, Member RAS Oncogene Family (RAB7A)-mediated fat phagocytosis increases intracellular PUFA production, thereby enhancing RSL3-induced ferroptosis in liver cancer cells. In contrast, lipid storage mediated by the tumour protein D52 (TPD52) may limit ferroptosis by isolating toxic oxidized lipids (Bai et al. 2019). Exogenous PUFAs induce formation and accumulation of LDs, leading to an increase in lipid ROS and ferroptosis in tumour cells (Dierge et al. 2021). Fas-associated factor family member 2 (FAF2) is a molecule that regulates LD formation and in vivo balance and is downregulated during orlistat-induced ferroptosis in cancer cells, supporting the anti-ferroptosis effect of LD (Zhou et al. 2021a).

### Nucleus

Release of nuclear DNA (HMGB1) is closely related to ferroptosis (Wen et al. 2019). Several DNA damage response pathways, such as the TP53, ataxia telangiectasia mutation (ATM), and FA complementation group D2 protein (FANCD2) pathways, play crucial roles in inhibiting or promoting ferroptosis. For example, Tumor protein 53 (TP53) activation can promote ferroptosis by downregulating SLC7A11 in breast cancer cells (Jiang et al. 2015). The absence of TP53 can trigger ferroptosis by activating the dipeptidyl peptidase 4 (DPP4)-dependent NADPH Oxidases (NOX) pathway in colon cancer cells (Xie et al. 2017). FA group D2 protein (FANCD2)-mediated DNA repair inhibits erastin-induced ferroptosis in bone marrow cells (Song et al. 2016). The iron-binding protein pirin limits autophagy-dependent ferroptosis by retaining HMGB1 in the nucleus (Hu et al. 2021a). In contrast, translocation of lysosomal CTSB or mitochondrial apoptosis-inducing factor mitochondria-associated 1 (AIFM1) to the nucleus can lead to local damage and induce ferroptosis (Kuang et al. 2020; Neitemeier et al. 2017). Therefore, translocation of different proteins in the nucleus affects the susceptibility of cells to ferroptosis.

## Mechanism of ferroptosis (Fig. 4)

### Iron regulation

As a cofactor, iron is involved in various biochemical processes, such as erythropoiesis, DNA synthesis and repair, oxidative phosphorylation, mitochondrial function and various enzymatic reactions. Under physiological conditions, duodenal epithelial cells absorb dietary iron, macrophages recover haemoglobin iron from senescent erythrocytes, and liver cells store iron. Normally, the balance of iron involves a balance between iron absorption, output, utilization and storage. When ferroptosis occurs, there is an excessive amount of free Fe^2+^ in the cell, and the extremely strong oxidation of Fe^2+^ could cause the Fenton reaction or Haber Weiss reaction with H_2_O_2_, thus generating hydroxyl radicals (OH^−^) and peroxides, which can lead to lipid peroxidation, damage the cell membrane and organelle membranes and lead to cell death. Sources of iron can be roughly divided into the following groups: 1) Controlling input of iron is one of the main ways to regulate the capacity of intracellular iron pools. 2) Synthesis of unstable iron from iron pools into various ferritin proteins is an important pathway for application of intracellular iron ions. 3) Recycling of iron ions by degrading intracellular ferritin proteins is also a way to increase the capacity of unstable iron pools in cells. 4) Transporting iron from cells to the outside is also a way to control and maintain cellular iron homeostasis (Li et al. 2020a).

**Fig. 4:**
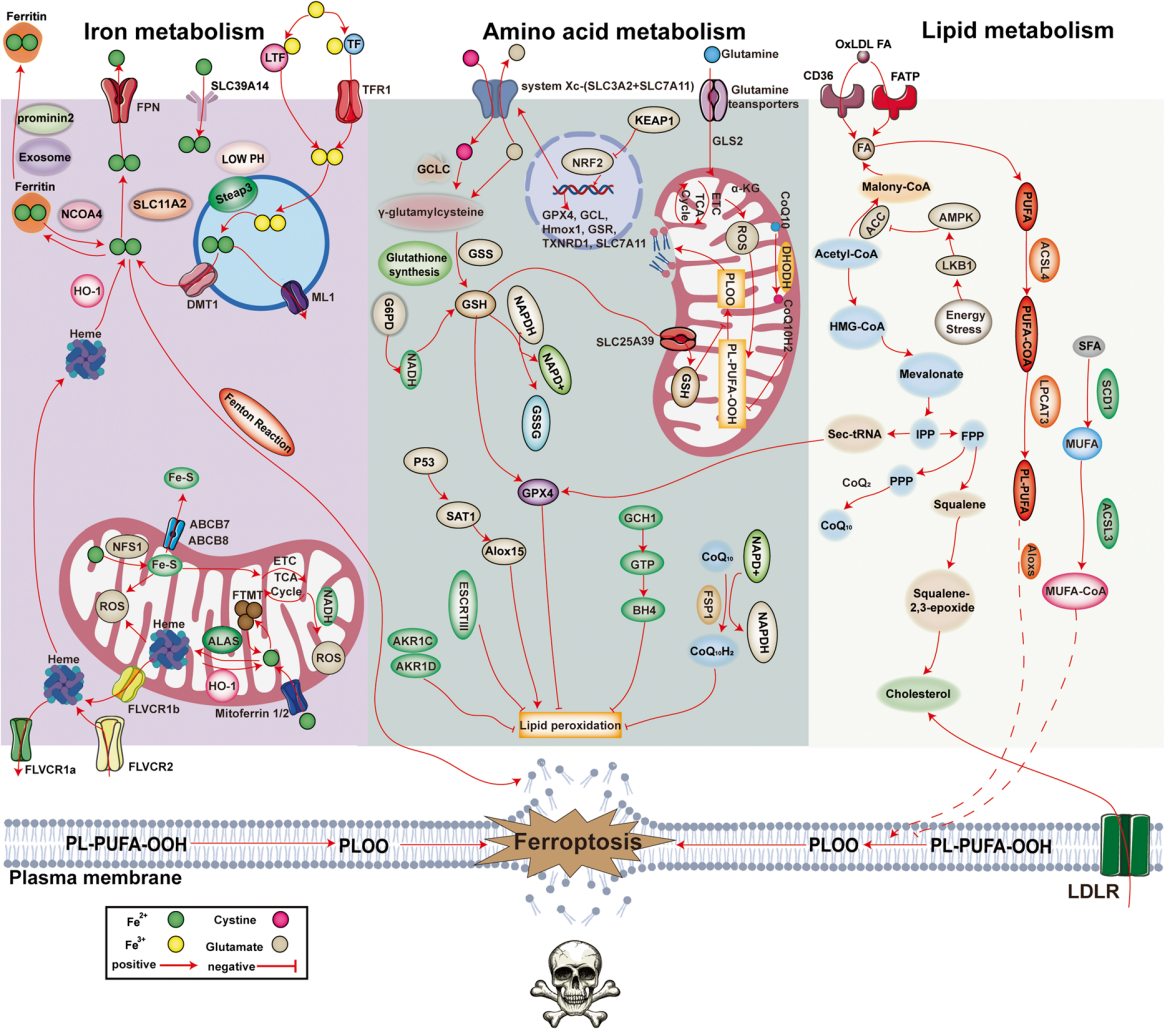
The mechanism of ferroptosis includes iron metabolism, amino acid metabolism, and lipid metabolism. GPX4: Glutathione peroxidase 4, NCOA4: Nuclear receptor coactivator 4, CoQ_10_: Coenzyme Q10, DMT1, Divalent metal transporter 1, FPN: Ferroportin, FSP1: Ferroptosis-suppressor protein 1, ESCRT III: Endosomal sorting complex required for transport III, BH4: Tetrahydrobiopterin, STEAP3: Steap3 metalloreductase, GCH1: GTP Cyclohydrolase 1, HO-1: Haem oxygenase 1, TF: Transferrin, TFR1: Transferrin receptor 1, ALOXs: Arachidonate lipoxygenase-s, ACSL3/4: Acyl-CoA synthetase long chain family member 3/4, FTMT, Mitochondrial ferritin, ROS: Reactive oxygen species, SLC7A11: Solute carrier family 7 member 11, Se: Selenium, SLC3A2: Solute carrier family 3 member 2, GSH: Glutathione, LPCAT3: Lysophosphatidylcholine acyltransferase 3, ETC: Electron transport chain, FLVCR1B: Feline leukaemia virus subgroup C receptor-related protein 1B, NFS1: Cysteine desulfurase, mitochondrial, ML1: Mucolipin 1, SLC11A2: Solute carrier family 11 member 2, FLVCR1a: Feline leukaemia virus subgroup C receptor-related protein 1a, FLVCR2: Feline leukaemia virus subgroup C receptor-related protein 2, ALAS: Aminolevulinic acid synthase, Fe–S: Iron–sulfur, ABCB7: ATP-binding cassette subfamily B member 7, ABCB8: ATP-binding cassette subfamily B member 8, SLC39A14: Solute carrier family 39 member 14, LTF: Lactotransferrin, SCD1: Stearoyl-CoA desaturase, NRF2: Nuclear factor erythroid 2-related factor 2, KEAP1: Recombinant kelch-like ECH-associated Protein 1, TCA cycle: Tricarboxylic acid cycle, GCL: Cysteine ligase, GLS2: Glutaminase 2, GSR: Glutathione reductase, TXNRD1: Glutathione reductase 1, SAT1: Spermidine/spermine N1-acetyltransferase 1, Alox15: Arachidonic acid 15-lipoxygenase-1, FA: Fatty acid, PUFA: Polyunsaturated fatty acid, AMPK: AMP-activated protein kinase, LKB1: Liver kinase B1, SFA: Saturated fatty acid, MUFA: Monounsaturated fatty acid, IPP: Isopentenyl pyrophosphate, FPP: Farnesyl diphosphate, PPP: Phytyl pyrophosphate, FATP: Fatty acid transport protein, ACC: Adrenocortical carcinoma, GSSG: Glutathione disulfide

The TFR correlates positively with the occurrence of ferroptosis. TFR transfers Fe^2+^ from the blood to the cell surface, after which Fe^2+^ enters cells through endocytosis. First, Fe^3+^ interacts with TF, is transported by transferrin receptor 1 on the cell membrane surface and then enters the cell via endocytosis, but the cell has a slightly acidic environment. With increasing pH, Fe^3+^ and TF are separated. Next, Six-Transmembrane Epithelial Antigen of Prostate 3 (STEAP3) returns Fe^3+^ to Fe^2+^, which enters the cytoplasm from lysosomes through Divalent metal transporter 1 (DMT1). Excess iron is stored in plasma ferritin in an oxidation‒reduction-inactive state, and a small amount of Fe^2+^ results in an unstable iron pool. Fe^2+^ can also be oxidized to Fe^3+^, and Fe^3+^ can leave the cell through ferritin (Fang et al. 2023).

Ferritin contains most of the iron atoms in the body. It is a complex composed of 24 subunits. The complex is composed of ferritin heavy chain (FTH) and ferritin light chain (FTL). Among them, FTH1 has iron oxidase activity, oxidized Fe^2+^ is stored as the more stable Fe^3+^, and FTL1 is stored in the iron pool as an endogenous iron chelating agent by binding with Fe^2+^. Under normal physiological conditions, excess Fe^2+^ in cells is oxidized to Fe^3+^ by the FTH of ferritin and is stored in ferritin, or excess Fe^2+^ is transported out of cells by Ferroportin1 (FPN1) on the cell membrane. When the iron content in cells is low, nuclear receptor coactivator 4 (NCOA4) interacts with FTH to mediate the autophagic degradation of ferritin. This process is called iron autophagy. Autophagic degradation of ferritin promotes release of Fe^3+^, which is subsequently converted to Fe^2+^ in cells. However, overactivation of iron-related autophagy leads to iron overload in cells and increases the sensitivity of cells to ferroptosis, resulting in depletion of glutathione and a reduction in GPX4, which is the most important ROS scavenging compound in the body, ultimately leading to ferroptosis. A decrease in NCOA4 levels increases iron levels in cells, leading to the Fenton reaction and thus reducing sensitivity to ferroptosis. Overexpression of NCOA4 increases the level of iron use in cells, thus enhancing sensitivity to ferroptosis (Li et al. 2021a; Gozzelino and Soares 2014).

Next, unstable iron in the iron pool is synthesized into various ferritin-containing proteins, which is an important pathway for the application of intracellular iron ions. Free iron in cells is utilized by various physiological and biochemical functions via iron regulatory proteins (IRP1/2), including synthesis of various iron-binding proteins, such as ferritin, or the formation of ferritin as a storage iron. Research has shown that tristetraprolin is expressed under cellular iron deficiency conditions and can reduce synthesis of various iron-binding proteins, especially iron sulfide proteins, by degrading mRNA transcripts, maintaining the capacity of the cell's iron pool (Sato et al. 2018). Expression of iron responsive element binding protein 2 (IREB2) increases synthesis of heavy and light chains in ferritin, inducing formation of stable iron ions. In addition, recycling iron ions by degrading intracellular ferritin is one way to increase the capacity of unstable iron pools in cells. For example, the NCOA4 protein can release iron ions from ferritin through selective autophagy. NFR2-regulated HO-1 can catalyse haem degradation to produce ferrous ions (Chang et al. 2018). Finally, transporting iron from cells to the outside of the cell is also one of the ways to control and maintain cellular iron homeostasis. FPN and prominin2 can transport iron ions and ferritin, respectively, to the extracellular space through various pathways. A decrease in expression of prominin2 has been confirmed to promote the occurrence of ferroptosis (Brown et al. 2019). Direct use of iron chelators to remove unstable iron is also a commonly used method in ferroptosis research.

### Metabolic mechanism

Glutathione is a tripeptide composed of the amino acid residues glutamic acid, cysteine and glycine and is soluble in water. There are two forms of glutathione in the human body: reduced GSH and oxidized GSSG. Reduced GSH is the main antioxidant in the human body. GSH is involved not only in synthesis of the cofactor GPX4 but also in reduction of the lipid hydroperoxide (LOOH) and removal of free radicals. GPX4 can metabolize toxic peroxides into nontoxic hydroxyl compounds, thereby reducing formation of ROS and preventing iron-mediated death. GPX4 is the most important inhibitor of lipid peroxidase in cells and the core regulator of ferroptosis. Depletion of glutathione leads to inactivation of GPX4 and weakening of protection against lipid peroxidation in cells, which leads to ferroptosis (Yu et al. 2021).

System X_C_^−^ is a member of the amino acid transporter family and is composed of two subunits: SLC7A11 and Solute carrier family 3 member 2 (SLC3A2). This system promoted uptake of cystine and biosynthesis of GSH. To synthesize glutathione, cells absorb cysteine and glutamic acid from the extracellular space at a 1:1 ratio through the system X_C_^−^. Cysteine and glutamate are formed by the ATP-dependent cysteine glutamate ligase (GCL) γ-glutamyl cysteine, and γ-glutamyl cysteine and glycine are subsequently catalysed by glutathione synthetase (GSS) to form glutathione. The efficiency of glutathione synthesis is mainly limited by the concentration of cysteine. In addition, intracellular glucose generates a large amount of NADPH via the pentose phosphate pathway. NADPH is the main source of synthesized GSH in vivo. GSH is dynamically balanced with oxidized glutathione through catalysis by GPX4, and depletion of GPX4 in tissues or cells can cause oxidative damage or cell death in an iron-dependent and noniron-dependent manner. For example, erastin and P53 reduce intracellular cysteine levels by inhibiting system X_C_^−^ so that glutathione is rapidly consumed by H_2_O_2_ and OH^−^. A decrease in intracellular glutathione concentrations inhibits the activity of GPX4, leading to a sudden decrease in the ability of cells to resist lipid peroxidation, and these cells become vulnerable to ferroptosis (Yang et al. 2014).

### Lipid peroxidation

Lipid peroxidation refers to loss of hydrogen atoms in lipids by free radicals or lipid peroxidases, which leads to oxidation, breakage and shortening of lipid carbon chains and production of cytotoxic substances such as lipid free radicals and lipid hydroperoxides, ultimately leading to cell death. The Fenton reaction results in production of a large amount of ROS, and ROS can interact with PUFAs and phosphatidylethanolamine (PE) to induce lipid peroxidation, thus producing toxic substances such as 4-hydroxynonenal (4-HNE) and malonaldehyde (MDA) and causing ferroptosis. The two lipid peroxidation reactions of PUFAs and PE are explained below (Chen et al. 2021b).

PUFAs are the main component of phospholipids in the cell membrane and organelle membranes and play an important role in maintaining normal growth, differentiation, ageing and death in cells. Because of the diallyl hydrogen atom, PUFAs are easily affected by lipid peroxidation. Lipid peroxidation can destroy the fluidity and stability of the cell membrane and organelle membrane structure, leading to cell membrane rupture and death. Lipid peroxidation of PUFAs involves active oxygen substances such as hydroxyl radicals and hydrogen peroxide, which generate hydrogen atoms in PUFAs to produce lipid free radicals (lipid ROS, L-). Next, lipid free radicals can react with oxygen molecules to generate lipid peroxidation free radicals (LOO-). Lipid peroxides capture hydrogen atoms from other PUFAs to form lipid free radicals and LOOH. Lipid peroxidation radicals can undergo cascade reactions (Jiang et al. 2021).

PE is a kind of glycerophospholipid that is located mainly in the inner membrane of mitochondria. PE is a precursor of phosphatidylcholine and affects oxidative phosphorylation and mitochondrial quality control. The affinity of PE for free radicals is much lower than that of PUFAs. Therefore, several enzymes need to be oxidized before lipid peroxidation occurs. First, arachidonoyl (AA) and adrenoyl moieties (AdA) produce AA-CoA and AdA-CoA under the action of ACSL4. Then, the latter forms PE-AA/AdA with PE under catalysis of LPCAT3. PE-AA/AdA is easily oxidized by free radicals or arachidonic acid lipoxygenase to form the cytotoxic lipid PE-AA/AdA-OOH, thus promoting ferroptosis. Several studies have shown that inhibiting ACSL4, lyso-phosphatidylcholine acyltransferase-3 (LPCAT3) and lipoxygenase (LOX) can suppress the occurrence and development of ferroptosis (Fang et al. 2023).

### Ferroptosis inhibitory protein 1 (FSP1)-CoQ10-NAD(P)H pathway

As early as 1996, research identified apoptosis-inducing factor (AIF) (Susin et al. 1996). Research has shown that AIFM2 is a p53-inducible gene also known as p53-responsive gene 3 (PRG3) (Ohiro et al. 2002). AIFM2 has been renamed FSP1 and inhibits ferroptosis mediated by CoQ_10_. The N-terminal nutmeg motif of FSP1 attracts it to the plasma membrane. CoQ_10_H_2_ is a lipophilic, free radical-trapping antioxidant (RTA). Subsequently, FSP1 reduces CoQ_10_ to CoQ_10_H_2_ through its oxidoreductase activity, which exerts antioxidant effects. The FSP1-CoQ-NAD(P)H-axis exerts a cytoprotective effect by catalysing the CoQ_10_-mediated inhibition of ferroptosis (Doll et al. 2019). In addition, CoQ_10_ was shown to explain the interaction between the GSH-GPX4 pathway and the FSP1-CoQ-NAD(P)H-axis (Fang et al. 2023).

### GTP cyclohydrolase-1 (GCH1)-tetrahydrobiopterin (BH4) pathway

The GCH1-BH4 pathway has recently been reported to inhibit ferroptosis through its metabolites BH4 and dihydrobiopterin. Due to the oxidative degradation effect of BH4 on phospholipids containing two PUFA tails, it directly captures peroxidized lipid radicals and participates in CoQ_10_ synthesis (Fanet et al. 2021). A genomic screening study revealed the ferroptosis-suppressor gene GCH1. The rate-limiting enzyme GCH1 was found to be overexpressed in BH4 biosynthesis to protect mouse fibroblasts from the effects of ferroptosis induced by RSL3 and GPX4 inhibition. The GCH1-BH4 axis prevents ferroptosis by inhibiting lipid peroxidation and increasing the abundance of CoQ_10 (_Kraft et al. 2020). In addition, research has shown that its accumulation in primary tumours can effectively prevent membrane PUFA damage under oxidative stress conditions, thereby inhibiting ferroptosis (Garcia-Bermudez et al. 2019).

### Dihydroorotate dehydrogenase (DHODH)-dihydroubiquione (CoQH_2_) pathway

DHODHs can regulate the de novo biosynthesis of pyrimidine (Zhang et al. 2022a). Research has shown that DHODH-CoQ_10_, along with mitochondrial GPX4, plays a role in defence against mitochondrial iron removal. DHODH, a pyrimidine synthase, can reduce CoQ_10_ on the inner membrane of mitochondria to CoQ_10_H_2_. Especially during rapid inactivation of GPX4, the amount of DHODH significantly increases to promote generation of panthenol, neutralizing lipid peroxidation and preventing mitochondrial ferroptosis. GPX4 and DHODH complement each other in inhibiting mitochondrial lipid peroxidation (Mao et al. 2021). Research has also shown that targeting exosomes from DHODH and GPX4 enhances sorafenib-induced ferritic anaemia, thereby increasing the sensitivity of hepatocellular carcinoma (HCC) cells to sorafenib (Li et al. 2022a).

### P53 pathway

**Fig. 5:**
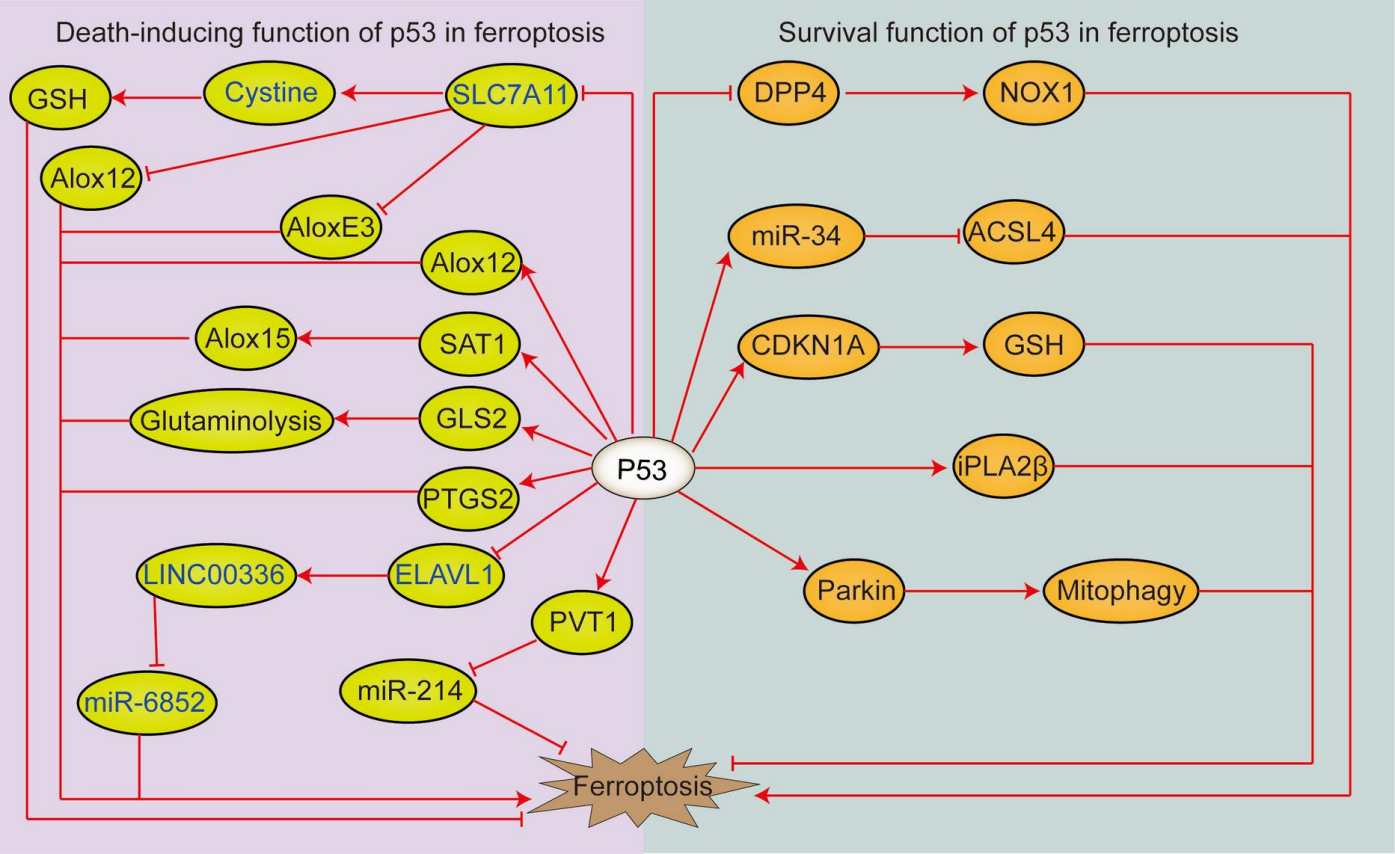
The dual role of p53 in the control of ferroptosis. ALOXE3: arachidonate lipoxygenase 3, ACSL4: acyl-coA synthetase long-chain family member 4, SLC7A11: solute carrier family 7 membrane 11, GSH: glutathione, DPP4: dipeptidyl peptidase-4, Alox12: arachidonic acid 12-lipoxygenase, Alox15: arachidonic acid 15-lipoxygenase, SAT1: spermidine/spermine N1-acetyltransferase 1, GLS2: glutaminase 2, PTGS2: Prostaglandin-endoperoxide synthase 2, ELAVL1: embryonic lethal abnormal visual-like protein, PVT1: Plasmacytoma variant 1, NOX1: Recombinant Nicotinamide Adenine Dinucleotide Phosphate Oxidase 1, CDKN1A: cyclin-dependent kinase inhibitor 1A, iPLA2β: group VIA calcium-independent phospholipase A2

Previous studies have shown that P53-induced ferroptosis is a double-edged sword (Kang et al. 2019)(Fig. 5).

### Death-inducing function of p53 in ferroptosis

1) Inhibition of SLC7A11 expression: Ubiquitination of ubiquitin-specific protease 22 (USP22) stabilizes expression of sirtuin 1 (SIRT1). In addition, SIRT1 overexpression can lead to p53 acetylation and decreased protein levels, whereas p53 inhibition can increase SLC7A11 levels (Ma et al. 2020). 2) Promotion of spermidine/spermine N1-acetyltransferase 1 (SAT1) expression: SAT1-induced ferroptosis requires arachidonic acid 15-lipoxygenase (Alox15), which is a lipoxygenase that catalyses AA peroxidation. SAT1 increases expression of Alox15, and Alox15 inhibitors rescue SAT1-induced ferroptosis (Ou et al. 2016). 3) Promotion of glutaminase 2 (GLS2) expression: GLS2 expression is induced in a p53-dependent manner in response to DNA damage or oxidative stress, and p53 is associated with the GLS2 promoter. An increase in GLS2 promotes glutamine metabolism and reduces intracellular ROS levels (Suzuki et al. 2010).

### Survival function of p53 in ferroptosis

1) Inhibition of dipeptidyl peptidase-4 (DPP4) activity: TP53 limits erastin-induced ferroptosis by blocking DPP4 activity in a transcription-independent manner. The absence of TP53 prevents nuclear accumulation of DPP4, thereby promoting membrane-related DPP4-dependent lipid peroxidation and ultimately leading to ferroptosis (Xie et al. 2017). 2) Promotion of cyclin-dependent kinase inhibitor 1A (CDKN1A/p21) expression: Ferroptosis sensitivity might be regulated by stress response-associated transcription factors and the tumour-suppressor protein p53. Researchers have shown that the stability of WT p53 can delay onset of ferroptosis caused by cystine deficiency. This delay requires the p53 transcriptional target CDKN1A (encoding p21) and is associated with slow intracellular consumption of GSH and reduced accumulation of toxic lipid ROS. Therefore, the p53-p21 axis may help cancer cells to cope with metabolic stress induced by cystine deficiency by delaying the occurrence of nonapoptotic cell death (Tarangelo et al. 2018).

### Hippo signalling pathway (Fig. 6)

For different cell lines (HCT116, H1650, PC9, and HepG2), as the cell density increases, the number of adhesion connections between cells and E-cadherin at the junction increases. E-cadherin can transmit cell density information to the E-cadherin-NF2- Yes associated protein (YAP)- YAP-transcriptional enhancer factor domain family member (TEAD)-ACSL4/TFRC pathway through Neurofibromin (NF2) (Wu et al. 2019). On the one hand, NF2 reduces degradation of large tumor suppressor homolog 1/2 (LATS1/2) by inhibiting the E3 ubiquitin ligase CRL4^DCAF1^(Li et al 2014). NF2 mediates phosphorylation of LATS1/2 by mammalian serine/threonine (Ste20) like kinases 1/2 (MST1/2) (Su et al. 2017; Li et al. 2015), which increases the activity of LATS1/2. Activated LATS1/2 phosphorylates YAP, promoting its cytoplasmic localization. Next, expression of the downstream genes ACSL4 and TFRC in YAP-TEAD is inhibited, and the sensitivity of cells to ferroptosis decreases (Wu et al. 2019).

**Fig. 6:**
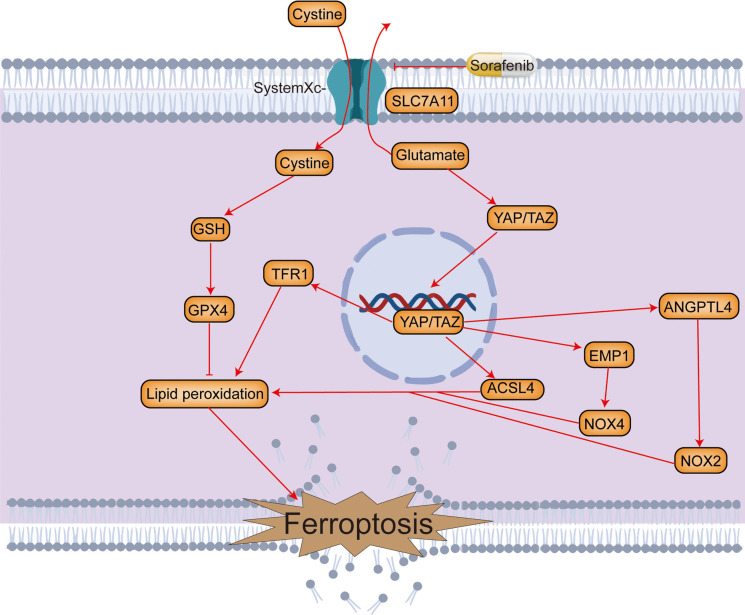
The role of hippo signalling pathway in ferroptosis. ACSL4: acyl-coA synthetase long-chain family member 4, SLC7A11: solute carrier family 7 membrane 11, TFR1: Transferrin Receptor 1, ANGPTL4: angiopoietin-like 4, NOX2: NADPH oxidases 2, NOX4: NADPH oxidase 4, EMP1: epithelial membrane protein 1, GSH: glutathione, GPX4: Glutathione peroxidase 4, YAP: Yes associated protein, TAZ: Recombinant Tafazzin

In contrast, BT474 cells exhibit low sensitivity to ferroptosis at both high and low cell densities, possibly due to high expression of E-cadherin at lower cell densities. However, MDA-MB-231 cells are highly sensitive to ferroptosis even at higher cell densities. This is because expression of E-cadherin in MDA-MB-231 cells remains extremely low even at high cell densities (Wu et al. 2019).

In addition, research has shown that the Hippo signalling pathway regulates ferroptosis through the epithelial membrane protein 1 (EMP1)-NADPH oxidase 4 (NOX4) pathway by affecting lipid peroxidation (Yang et al. 2019). Furthermore, the Hippo signalling pathway participates in glutamate metabolism in lung adenocarcinoma (Zhang et al. 2021a).

Finally, a series of recent reports have indicated that there are yet other mechanisms for suppressing ferroptosis, independent of glutathione/GPX4, FSP1/DHODH/CoQ10 and GCH1/BH4. Inhibiting NUFR1 promotes ferroptosis by downregulating expression of lipocalin 2 (LCN2), increasing the intracellular iron concentration, decreasing lipid peroxidation, and decreasing DNA oxidative damage. It can also stabilize the mitochondrial membrane and participate in maintaining mitochondrial function (Liu et al. 2021b; Tan et al. 2021). Second, during IL4i1 metabolism, indole-3-pyruvate is produced, which inhibits ferroptosis by eliminating free radicals and reducing the expression of genes related to ferroptosis (Zeitler et al. 2021). Glucose starvation actually inhibits ferroptosis, and this protective effect depends on the activity of the energy-sensing kinase AMPK. Therefore, when glucose deficiency occurs, AMPK is activated, initiating an energy stress protection program to combat ferroptosis, which involves impaired biosynthesis of the polyunsaturated fatty acids (PUFAs) necessary for lipid peroxidation-induced ferroptosis. Its mediating pathways include AMPK-NFR2, AMPK-mTOR, AMPK-JAK2/STAT3-P53-GPX4/ROS-AMPK-SCD1, LKB1-AMPK-ACC1, AMPK-SREBP1/SCD1, PP2A-AMPK-EEF2, and AMPK-BECN1-SLC7A11-Xc-(Dodson et al. 2019; Han et al. 2020a; Shen et al. 2020; Tang et al, 2021a; Li et al, 2020b; Zhao et al,^2020^; Zhong et al,^2020^; Song et al, 2018). HIF2α-HILPDA can also increase ferroptosis sensitivity (Zou et al. 2019).

## Ferroptosis in Different Cardiovascular Cells

### Endothelial cells

The vascular endothelium plays an important role in maintaining vascular function, regulating vascular permeability, controlling vascular contraction and relaxation, inhibiting vascular smooth muscle cell proliferation, platelet aggregation, etc. It has anti-thrombotic, anti-inflammatory, and antioxidant effects. Once vascular endothelial function is impaired, the lipids in the blood will slowly accumulate in the vascular wall, causing atherosclerosis in the blood vessels and subsequent plaque formation. Over the years, plaques become increasingly larger, and when the plaques fall off, they can form blood clots, causing vascular stenosis and blockage, leading to a series of cardiovascular and cerebrovascular diseases (Bloom et al. 2023). The ROS produced during oxidative stress can oxidize lipids and proteins, induce inflammatory reactions, directly damage vascular cells, and lead to endothelial dysfunction (Ardiana et al. 2021). Research has shown that miR-17–92 can protect HUVECs from erastin-induced ferroptosis, revealing a new mechanism by which the TNF-alpha-induced protein 3 (TNFAIP3)-ACSL4 axis is targeted to protect endothelial cells from erastin-induced ferroptosis (Xiao et al. 2019a). When endothelial cells are exposed to particulate matter 2.5 (PM2.5), their iron content increases, and lipid peroxidation subsequently leads to an increase in the cellular iron concentration and the secretion of inflammatory cytokines. This can be alleviated through lipid peroxidation inhibitors and iron chelating agents (Wang and Tang 2019). Mouse aortic endothelial cells (MAECs) treated with ox-LDL or erastin showed elevated levels of lipid peroxidation in damaged mitochondria. However, Fer-1 can reduce lipid peroxide levels, confirming that inhibiting ferroptosis can ameliorate ox-LDL-induced endothelial cell damage and lipid peroxidation (Bai et al. 2020).

### Vascular smooth muscle cells

Vascular smooth muscle cells (VSMCs) are the main cellular components that comprise the vascular mesomembrane and are important metabolic and endocrine organs in the body. They play an important role in various physiological processes. Vascular smooth muscle cell dysfunction is closely related to the occurrence and development of diseases. Research has shown that in pathological processes, vascular smooth muscle cells participate in hypertension through their own proliferation, migration, and synthesis of extracellular matrix. Physiological and pathological processes occur in various vascular diseases, such as atherosclerosis, transplant vascular disease, restenosis after angioplasty and repair of vascular wall injury (Owens et al. 2004).

Cigarette smoke extract (CSE)-induced cell death in rat VSMCs is completely inhibited by specific ferroptosis inhibitors and iron chelators. Moreover, CSE induces upregulation of PTGS2 mRNA expression, lipid peroxidation, and intracellular glutathione consumption. Moreover, CSE causes a loss of smooth muscle cells in isolated medial aortic vessels. These findings indicate that ferroptosis is the cause of CSE-induced vascular smooth muscle cell death (Sampilvanjil et al. 2020). Related studies have shown that metformin has an anti-ferroptotic effect on vascular calcification. Palmitic acid (PA) treatment upregulates expression of the extracellular matrix protein periodin (POSTN), which is an important negative regulator of p53 and leads to ferroptosis, in VSMCs. In addition, we found that metformin enhances the antioxidant capacity of VSMCs by activating Nrf2 signalling (Ma et al. 2021). Lipoprotein 2 significantly promotes ERS (upregulation of GRP78 and NOGO transcription, increased expression of SOD2, and slightly enhanced mitochondrial membrane potential) and proliferation (as evaluated by Ki67 staining and BrdU incorporation) in PH (pulmonary hypertension) while simultaneously increasing intracellular iron levels in human PASMC cells. Lipoprotein 2 can also reduce proliferation in ERS and VSMCs, and reducing ERS can alleviate progression of AS. FeSO4 treatment of human PASMCs induces similar ERS and proliferation responses, and the iron chelating agent deferoxamine eliminates the ERS and proliferation induced by Lcn2 in cultured human PASMCs (Wang et al. 2017).

### Macrophages

In apoE^−/−^ mice, a high-iron diet significantly enriched CD68. In addition, a high-speed iron diet strongly induces TGF-β (the transformation of growth factor β), TNF-α, IL-6, IL-23, IL-10, and IL-1β. Iron loading subsequently triggers polarization of macrophages towards the pro-inflammatory M1 phenotype. In addition, ferric ammonium citrate (FAC) promotes the polarization of M1 macrophages into bone marrow-derived macrophages (BMDMs) (Hu et al. 2019).

Iron deficiency enhances EMMPRIN expression, MMP-9 production, and MMP-9 enzymatic activity in THP-1-derived macrophages and foam cells. Iron deficiency induces activation of NF-κB and p38 MAPK. By using a p38 inhibitor and an NF-κB inhibitor, it was established that iron deficiency-induced induction of EMMPRIN and MMP-9 requires consecutive upstream activation of p38 MAPK and NF-κB. This pro-inflammatory action was not prevented by the PPAR-γ agonist. Moreover, iron deficiency did not modulate PPAR-γ expression. A retinal X receptor agonist suppresses the effects of iron deficiency on EMMPRIN, MMP-9, and NF-κB but not on MAPK activation. Iron deficiency enhances atheroma inflammation through the p38 MAPK-NF-κB-EMMPRIN/MMP-9 pathway (Fan et al. 2011). Furthermore, we discovered that inducible nitric oxide synthase (iNOS)/NO• enrichment of activated M1 (but not alternatively activated M2) macrophages/microglia modulates susceptibility to ferroptosis. Genetic or pharmacologic depletion/inactivation of iNOS confers sensitivity on M1 cells, and NO• donors promote the resistance of M2 cells to ferroptosis. In vivo, compared with M2 phagocytes, M1 phagocytes exhibit greater resistance to pharmacologically induced ferroptosis (Kapralov et al. 2020). Transcriptome differential gene expression analysis has reveals significant differences in expression of genes related to iron concentration after pretreatment with itaconic acid. 4-Octylitaconic acid (4-OI) is a cellular osmotic derivative of endogenous itaconic acid that can significantly reduce lung injury, increase LPS-induced GPX4 levels, and reduce PTGS2, MDA, and lipid ROS. In vitro experiments have shown that both 4-OI and Fer-1 can inhibit LPS-induced lipid peroxidation and damage in THP-1 macrophages. Mechanistically, we found that 4-OI inhibits GPX4-dependent lipid peroxidation by increasing the accumulation and activation of Nrf2. Silencing Nrf2 eliminates the inhibitory effect of 4-OI on the iron concentration in THP-1 cells. In addition, the protective effect of 4-OI on ALI is eliminated in Nrf2 gene knockout mice (He et al. 2022).

### Cardiomyocytes

When the iron content exceeds the normal level in the body and is excessively deposited in the body, structural damage and dysfunction of the heart can occur (Fang et al. 2023). ZJ01 triggers Nrf2 nuclear translocation in vitro, subsequently resulting in increased mRNA levels of the Nrf2 target genes HO-1 and NQO1. Moreover, ZJ01 suppresses LPS-induced production of ROS and mRNA levels of the pro-inflammatory cytokines TNF-α, IL-1β and IL-6 in H9c2 cardiomyocytes. In an in vivo mouse model of septic cardiomyopathy induced by intraperitoneal injection of lipopolysaccharide, ZJ01 showed a cytoprotective effect, upregulated Nrf2 protein nuclear accumulation, and markedly suppressed the abovementioned cytokine levels in cardiomyocytes (Jiang et al. 2018). We examined the cardioprotective effect of exogenous spermine on DCM in streptozotocin (STZ)-induced T1D rats and high-glucose (HG)-incubated neonatal rat cardiomyocytes. Exogenous spermine significantly attenuated cardiac dysfunction in T1D rats, as characterized by improved echocardiography, decreased fibrosis, reduced myocardial ERS and oxidative stress, and increased expression of myocardial membrane CaSR. In cultured neonatal rat cardiomyocytes, exogenous spermine attenuated myocardial injury induced by HG treatment, as demonstrated by restored cellular glucose uptake capacity, reduced expression of apoptotic markers, decreased levels of oxidative stress, ERS and the unfolded protein response, and upregulated cell membrane CaSR. Mechanistically, the cardioprotective effect of spermine appears to be dependent upon effective elimination of ROS and upregulation of CaSR expression through suppression of the Nrf2-ROS-p53-MuRF1 axis. Taken together, these results suggest that exogenous spermine protects against DCM in vivo and in vitro, partially by suppressing ROS and p53-mediated downregulation of cell membrane CaSR (Wang et al. 2020a).

We further report a novel mechanism of Nrf2-mediated myocardial damage in type 1 diabetes (T1D) patients. Global Nrf2 knockout (Nrf2KO) negligibly affected the onset of cardiac dysfunction induced by T1D but slowed cardiac dysfunction progression in mice independent of sex. In addition, Nrf2KO inhibited cardiac pathological remodelling, apoptosis, and oxidative stress, which are associated with both the onset and progression of cardiac dysfunction in T1D patients. This Nrf2-mediated progression of diabetic cardiomyopathy was confirmed by a cardiomyocyte-restricted (CR) transgenic approach in mice. Moreover, cardiac autophagy inhibition via CR knockout of the autophagy-related 5 gene (CR-Atg5KO) led to early onset and accelerated development of cardiomyopathy in T1D, and CR-Atg5KO-induced adverse phenotypes were rescued by additional Nrf2KO. Mechanistically, chronic T1D leads to glucolipotoxicity, inhibiting autolysosome efflux, which in turn intensifies Nrf2-driven transcription to fuel lipid peroxidation while inactivating Nrf2-mediated antioxidant defence and impairing Nrf2-coordinated iron metabolism, thereby leading to ferroptosis in cardiomyocytes (Zang et al. 2020).

### Main detection methods for ferroptosis (Table 2)

## Ferroptosis in cardiovascular diseases

### Myocardial infarction

Myocardial infarction (MI) is the leading cause of death worldwide. The common pathophysiology of MI includes abnormal supplementation of myocardial oxygen or coronary atherothrombosis. When atherothrombosis occurs, platelets aggregate and block the coronary artery, leading to myocardial ischaemia and necrosis. On the other hand, a lack of nutrients and oxygen leads to inflammatory reactions and cardiomyocyte death. It is now clear that ferroptosis is both a pro-inflammatory reaction and an RCD (Wang and Kang 2021). The probability of premenopausal women having coronary artery disease (CAD) is far less than that of men. Iron accumulates with age, and the accumulated amount of iron in postmenopausal women reaches the level of that in men (Sullivan 1981).

**Table 2:** Main detection methods for ferroptosis

Experimental methods	Purpose	Advantage	Disadvantage
Transmission electron microscopy	Detecting morphological feature of ferroptosis	Observing the morphological features of ferroptosis	Subjectivity, difficult to operate
CCK-8 assay	Detecting cell death based on chemiluminescence reaction	Easy to operate and low equipment requirements	Not specific for detecting ferroptosis
LDH release assay	Detecting cell death based on chemiluminescence reaction	Easy to operate and low equipment requirements	Not specific for detecting ferroptosis
Immunohistochemical staining for lipid peroxidation	Detecting MDA and 4-HNE protein of lipid peroxidation	Easy to operate and achieve quantitative MDA and 4-HNE protein	Not specific for detecting ferroptosis
MDA and 4-HNE assay	Detecting MDA and 4-HNE product of lipid peroxidation	Easy to operate and achieve quantitative MDA and 4-HNE protein	Not specific for detecting ferroptosis
Propidium iodide	Detecting cell death based on chemiluminescence reaction	Easy to operate and low equipment requirements	Not specific for detecting ferroptosis
Western blot for key regulators for ferroptosis	Detecting key regulators of ferroptosis	Strong specific for detecting key regulators of ferroptosis	Subjectivity, prone to false positives
Quantitative real-time PCR for key regulators for ferroptosis	Detecting key regulators of ferroptosis	Strong specific for detecting key regulators of ferroptosis	Subjectivity, prone to false positives
SYTOX Green	Detecting cell death	Easy to operate and achieve quantitative	Not specific for detecting ferroptosis
C11-BODIPY^581/591^	Detecting lipid ROS in living cells	Detecting and quantifying lipid peroxidation in the membrane of living cells	Not specific for detecting lipid peroxidation
2′,7′-dichlorodihydrofluorescein diacetate staining	Exploring cytoplasmic ROS	Easy to operate and low equipment requirements	Not specific for detecting ferroptosis
Dihydroethidium staining	Detecting ROS in living cells and paraffin slices	Easy to operate and low equipment requirements	Not specific for detecting ferroptosis
Liperfluo	Detecting lipid ROS in living cells	Detecting and quantifying lipid peroxidation in the membrane of living cells	Not specific for detecting lipid peroxidation
Fe^2+^ assay	Detecting total iron level	Accurate determination of iron levels	Only for total iron level detection
Prussian blue staining	Detecting the distribution and amount of iron deposit in tissues	Detecting and quantifies free iron in the paraffin slices	Not suitable for detecting free iron levels in living cells
RhoNox-1	Detecting Fe^2+^ level in living cells	Detecting and quantifies Fe^2+^ in the membrane of living cells	Not suitable for paraffin slices
FerrOrrange	Detecting Fe^2+^ level in living cells	Detecting and quantifies Fe^2+^ in the membrane of living cells	Not suitable for paraffin slices
Mito-FerroGreen	Detecting Fe^2+^ level in mitochondria	Detecting and quantifies Fe^2+^ in the mitochondria of living cells	Not suitable for paraffin slices
FRET iron probe 1	Detecting Fe^2+^ level in living cells	Detecting and quantifies Fe^2+^ in the membrane of living cells	Not suitable for paraffin slices
GSH/GSSH assay kit	Detecting GSH and GSH/GSSG content	Easy to operate and low equipment requirements	Not specific for detecting ferroptosis

*ROS* reactive oxygen species, *MDA* malonaldehyde, *4-HNE* 4-hydroxynonenal, *GSH* glutathione, *GSSG* glutathione disulfide

According to clinical research, in a cohort study of 12,033 patients, an increase in the serum ferritin concentration was related to the presence of early coronary atherosclerosis markers, independent of traditional cardiovascular risk factors (Sung et al. 2012). A prospective study revealed that a 9-year follow-up of cardiac magnetic resonance (CMR) in 74 ST-segment elevation myocardial infarction (STEMI) patients revealed persistent iron and oedema in the infarct core within ten years after STEMI, which was associated with initial infarction severity and poor infarction healing. This finding suggested that persistent Fe^2+^ deposition in the infarcted core leads to persistent myocardial oedema, and iron overload and that the lipid peroxidation caused by ferroptosis correlates positively with coronary artery risk (Mayr et al. 2022). In a prospective clinical trial, ninety-eight patients (14 females, 84 males, mean age: 57.7 years) with MI reperfused with percutaneous coronary intervention (PCI) were studied within the first week (1 W) and at 4 months (4 M) after the event. Magnetic resonance imaging (MRI) can be used to identify MI, myocardial bleeding, and microvascular occlusion (mvO), as well as to measure left ventricular volume, function, and quality. Intramyocardial haemorrhage (IMH) and myocardial iron residue are independent predictors of left ventricular remodelling after ST-segment elevation MI (Ganame et al. 2009). Research has shown that proteomics can be used to analyse coronary arteries and normal coronary arteries in patients with CAD. Disease-related proteins can be detected by measuring expression levels of proteins between the control and disease status. Expression of ferritin optical chains in the affected branches of coronary arteries in patients with CAD significantly increases, but expression of ferritin optical chain mRNA decreases, indicating that increased expression of ferritin optical chains in coronary arteries may be related to increased protein stability or regulation of expression in diseased tissues. There is a correlation between excessive iron storage and a high risk of CAD (You et al. 2003). In a prospective study, cardiovascular coronary revascularization was performed on 48 patients with ST-segment elevation myocardial infarction 4 ± 2 days after initial magnetic resonance imaging. Among them, 40 of the patients underwent follow-up scans at 5 ± 2 months, and magnetic resonance imaging of ST segment elevation in MI patients after percutaneous reperfusion treatment revealed an increase in iron content in the infarcted area, indicating that myocardial iron in the infarcted area is a risk factor for left ventricular remodelling after MI (Bulluck et al. 2016).

In contrast, a Mendelian randomization study confirmed the hypothesis that higher iron status reduces CAD risk (Gill et al. 2017). Another meta-analysis revealed a negative correlation between transferrin levels and CAD with high transferrin saturation and a reduced risk of CAD/MI (Das et al. 2015).

Proteomic analysis of infarcted mice and control mice revealed that the protein level of GPX4 decreased during MI. In vivo and in vitro experiments have confirmed that knocking down GPX4 exacerbates myocardial injury in MI and that inhibiting expression of GPX4 increases the sensitivity of cardiomyocytes to ferroptosis when cysteine is depleted (Park et al. 2019). HIP-55 is the substrate of AKT kinase and is phosphorylated by AKT at the S269/T291 site. Phosphorylated HIP-55 recruits 14–3-3τ to form HIP-55/14–3-3τ, and the resulting complex inhibits the MAP4K1/JNK/GPX4 ferroptosis pathway, producing cardioprotective effects after MI (Jiang et al. 2023). Research has shown that inhibiting ferroptosis can delay ventricular remodelling in MI mice, thereby protecting cardiomyocytes (Fang et al. 2019). MI induces platelet internalization, resulting in release of miR-223-3p, a platelet-enriched miRNA. By targeting ACSL3, miR-223-3p delivered by internalized platelets causes a reduction in stearic acid-phosphatidylcholine in cardiomyocytes. The presence of stearic acid-phosphatidylcholine protects cardiomyocytes against ferroptosis (Miao et al. 2023).

### Atherosclerosis

Atherosclerosis (AS) is characterized by lipid metabolism disorder and is associated with smooth muscle proliferation, endothelial dysfunction, apoptosis, necrosis, inflammation and formation of foam cells and lipid plaques. Ferroptosis is a pro-inflammatory reaction that is closely related to AS, and AS is strongly linked to changes in iron levels in the body. Excess iron accumulation during ferroptosis catalyses the Fenton reaction to produce lipid ROS, which oxidize low-density lipoprotein to form oxidized low-density lipoprotein, leading to lipid deposition, foam cell formation, and endothelial dysfunction. When endothelial function is impaired, vascular endothelial white blood cell adhesion factors and inflammatory factors are excessively secreted for a long time, resulting in generation of new blood vessels in the centre of the plaque, increasing plaque vulnerability and causing bleeding within the plaque, ultimately leading to the occurrence of AS (Martinet et al. 2019). In addition, iron overload leads to mitochondrial damage in endothelial cells through the ROS and cyclooxygenase pathways, affects the inflammatory phenotype of macrophages, and promotes formation of early AS. Iron-catalysed free radical reactions can cause oxidation of low-density lipoprotein in endothelial cells, smooth muscle cells or macrophages, which may be risk factors for the formation of atherosclerotic lesions. Moreover, formation of foam cells and iron overload are generally considered risk factors for AS. Because iron ions can promote oxidative stress and inflammation, free radicals generated by iron ions not only causes endothelial cell apoptosis but also oxidizes LDL to promote macrophage phagocytosis and formation of foam cells, accelerating progression of AS (Sullivan 1981; Xiao et al. 2020).

Prospective results from the Bruneck study showed that an increase in the serum ferritin concentration is a risk factor for progression of carotid AS after 5 years of ultrasonic follow-up (Kiechl et al. 1997). In ApoE^−/−^ mice fed an HFD, AS may lead to ferroptosis, and inhibiting the iron concentration can prevent lipid peroxidation and worsening of AS in the thoracic aorta. Furthermore, inhibiting iron concentrations can inhibit lipid peroxidation and low-density lipoprotein-induced dysfunction of small arterial endothelial cells, reverse cell viability, inhibit ferroptosis, and inhibit progression of arteriosclerosis. Studies have shown that a high iron diet increases the expression level of inflammatory factors TGF-β, TNF-α, IL-6, IL-23, IL-10, and IL-1β. It was found that glycolysis is involved in the polarization of M1 macrophages triggered by iron load, and finally iron load accelerated the progression of AS by inducing inflammation and enhancing glycolysis of macrophages (Hu et al. 2019). Compared with those in apolipoprotein E^−/−^ mice, which have normal ferruginous acid levels, AS in iron-loaded ApoE^−/−^ FPNwt/C326S mice were seriously aggravated, suggesting that iron can promote AS. Iron deposition in the middle layer of arteries is related to plaque formation, vascular oxidative stress, and dysfunction. A low-iron diet and ferroptosis inhibitor strongly improved endothelial dysfunction in ApoE^−/−^ FPNwt/C326S mice (Vinchi et al. 2020). Expression of the anti-ferroptosis-related genes SLC7A11 and GPX4 was significantly reduced in the human umbilical vein endothelial cell (HUVEC) injury model, yet expression of the anti-ferroptosis-related genes SLC7A11 and GPX4 was significantly increased in the ferroptosis inhibitor treatment group. Finally, ferroptosis is involved in endothelial dysfunction, and activation of the p53-xCT-GSH axis plays a critical role in endothelial cell ferroptosis and endothelial dysfunction (Luo et al. 2021a). Related case‒control studies from France have confirmed that the serum ferritin concentration is significantly greater in patients with AS than in patients without AS, and for every 10 μg/L increase in the serum ferritin concentration, the risk of AS increases by 3% (Ahluwalia et al. 2010). Finally, studies have shown that increased expression of Jak2V617F in the circulation promotes erythropoiesis and ROS production in red blood cells, leading to increased endothelial permeability, inflammatory cell infiltration, and increased entry of red blood cells into necrotic areas, ultimately resulting in increased phagocytosis and ferroptosis and elevating the volume of the necrotic core in the lesion area. Liproxstatin-1, a ferroptosis inhibitor, reduces the area of AS plaques (Liu et al. 2022).

Due to the impact of iron overload on the absorption and transport balance of fatty acids in the liver, a study showed that increasing iron intake resulted in a decrease in serum total cholesterol triglyceride and LDL levels, ultimately leading to a decrease in the number of arterial plaques in ApoE^−/−^ mice (Xiao et al. 2021).

### Ischaemia/reperfusion (I/R) injury (Fig. 7)

When IR occurs, the coronary artery blood supply cannot meet the needs of the myocardium, which leads to metabolic disorders in cardiomyocytes and death in cardiomyocytes, resulting in changes in cardiac structure and function. I/R causes reperfusion-related oxidative damage, which is associated with lipid peroxidation and increased intracellular iron levels (Li et al. 2021c). Most current studies investigating the role of ferroptosis in MIRI have focused mainly on ERS, ROS production, GPX-4, and the autophagy-dependent ferroptotic pathway.

Magnetic resonance imaging (MRI) was performed on 48 patients with ST-segment elevation MI at 4 days and 5 months after PCI, and the residual iron levels in the infarcted area and left ventricular remodelling area were relatively high (Ooko et al. 2015). Studies have shown that during myocardial ischaemia in mice, there is no significant change in the average levels of ACSL4, GPX4, iron, or malondialdehyde, which are related to ferroptosis. However, after myocardial reperfusion, ACSL4, iron, and malondialdehyde levels increase, but GPX4 levels decrease. Additionally, when TFR1 is activated, ferritin autophagy increases, leading to iron deposition in cells (Tang et al. 2021b; Fan et al. 2021a).

Alox15 and its metabolite 15-HpETE are key factors triggering ferroptosis, and 15-HpETE can promote binding of PGC1-α to the ubiquitin ligase RNF34 to promote its degradation and weaken mitochondrial biogenesis, ultimately leading to an increase in levels of mitochondrial lipid peroxidation products, dysfunction, and morphological abnormalities. Moreover, cardiomyocytes exhibit a unique pattern of ferroptosis (Cai et al. 2023). Bioinformatics analysis of related studies has revealed that the three molecules USP7, p53 and TFR1 form a unique USP7/p53/TFR1 pathway. USP7, p53 and TFR1 are upregulated in I/R-treated mice. Inhibiting USP7 activates p53 by inhibiting deubiquitination, leading to downregulation of TFR1 and activation of ferroptosis. Knockout of TFR1 inhibited H/R-induced ferroptosis, but p53 was not deubiquitinated. Therefore, a new USP7/p53/TFR1 pathway was found in rat hearts after I/R, in which upregulation of USP7 promoted ferroptosis by activating the p53/TFR1 pathway (Tang et al. 2021c).

**Fig. 7:**
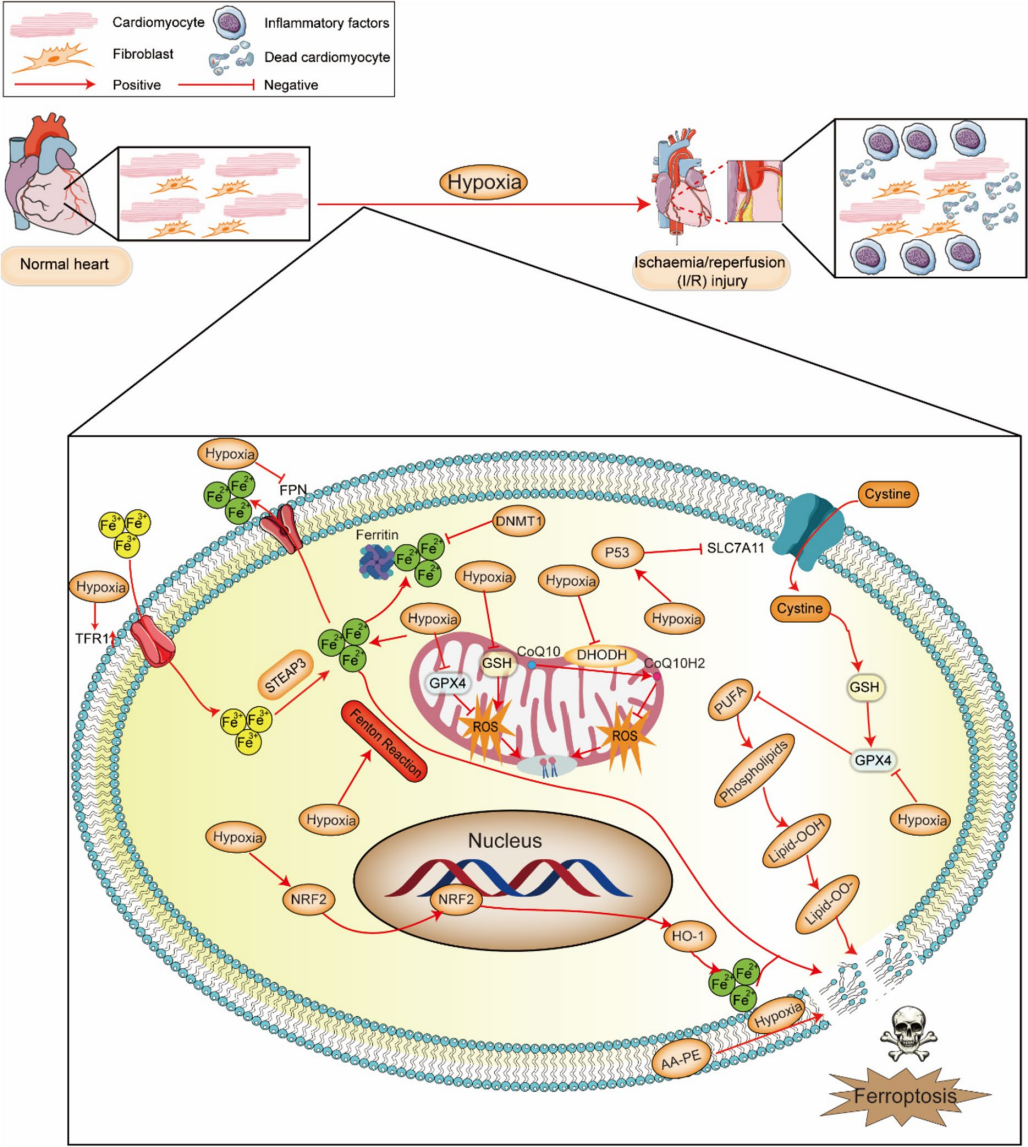
The role of ferroptosis mechanism in Ischaemia/reperfusion (I/R) injury. SLC7A11: solute carrier family 7 membrane 11, TFR1: Transferrin Receptor 1, GSH: glutathione, GPX4: Glutathione peroxidase 4, PUFA: polyunsaturated fatty acids, DHODH: Dihydroorotate dehydrogenase, ROS: reactive oxygen species, NRF2: NF-E2-related factor 2, FPN: Ferroportin, STEAP3: Six-Transmembrane Epithelial Antigen of Prostate 3, HO-1: heme oxygenase 1, DNMT1: DNA methyltransferase 1, AA-PE: arachidonic acid-phosphatidylethanolamines, CoQ10: Coenzyme Q10, CoQ10H2: reduced coenzyme Q10

### Hypertrophic cardiomyopathy

Hypertrophic cardiomyopathy is a common inherited CVD present in one in 500 individuals in the general population. It is caused by more than 1400 mutations in 11 or more genes encoding proteins of the cardiac sarcomere. Although hypertrophic cardiomyopathy is the most common cause of sudden death in young people (including trained athletes) and can lead to functional disability from heart failure and stroke, the majority of affected individuals are undiagnosed, and many people do not experience greatly reduced life expectancy or substantial symptoms (Maron and Maron 2013).

Feeding mice (ferritin H, Fth^−/−^) a high-iron diet resulted in severe heart injury and hypertrophic obstructive cardiomyopathy, which are typical molecular characteristics of ferroptosis and included increased levels of GSH and lipid peroxidation. Ultimately, Fth^−/−^ cardiomyocytes exhibited reduced expression of the anti-ferroptosis gene SLC7A11, and selectively overexpressing SLC7A11 in cardiomyocytes increased GSH levels and prevented cardiac ferroptosis (Fang et al. 2020).

xCT acts as a suppressor of AngII-mediated cardiac hypertrophy by blocking ferroptosis. Positive modulation of xCT may therefore represent a novel therapeutic approach against cardiac hypertrophic diseases. The ferroptosis inhibitor Fer-1 can inhibit the above processes (Zhang et al. 2022b). Microarray data analysis exploring ferroptosis related gene therapy have revealed targets associated with HCM (Wang et al. 2022a). Friedrich's ataxia (FRDA) is an HCM caused by mutations in the FXN gene. Studies have shown that the ferroptosis inhibitor SRS11-92 can reduce cell death in fibroblasts derived from primary FRDA patients and in mouse fibroblasts with FRDA-related mutations (Cotticelli et al. 2019).

### Iron overload cardiomyopathy

Pathological and physiological conditions such as thalassemia and sickle cell disease, chronic liver disease, Friedreich ataxia, delayed skin porphyria, excessive dietary iron intake, and other rare diseases that lead to abnormal iron metabolism, such as congenital transferrin deficiency, in addition to recurrent transfusion, ineffective haematopoiesis, peripheral haemolysis, and increased gastrointestinal iron absorption, are reasons for iron overload in primary and secondary haemochromatosis patients (including α, β). Iron overload in cardiomyocyte leads to myocardial dysfunction, which is known as iron overload cardiomyopathy (Farmakis et al. 2017; Zhang et al. 2019).

Under iron overload, L-type and T-type calcium channels are involved in myocardial iron uptake, and lipid-carrying protein-2 (LCN-2) and its receptors have been found to be involved in this process in recent studies (Kumfu et al. 2011; Oudit et al. 2003). Excessive free iron in cardiomyocyte can damage cardiomyocytes through various mechanisms. First, the Fenton reaction can lead to an increase in ROS, damaging the cell membrane and organelle membrane. ROS also slow inactivation of calcium ion channels, leading to an increase in calcium ion influx and causing myocardial diastolic dysfunction. When iron overload intensifies, iron ions and calcium ions compete through calcium channels, leading to a decrease in calcium ion influx and causing contractile dysfunction. Iron ions can also enter mitochondria, causing mitochondrial dysfunction through oxidative stress, leading to disordered mitochondrial energy metabolism (Kremastinos and Farmakis 2011).

We report that the anti-RA drug auranofin has dual effects: increasing hepcidin expression via the NF-κB/IL-6/STAT3 signalling pathway and at high doses, inducing ferroptosis by inhibiting the thioredoxin system. These findings provide compelling evidence that AUR may serve as a novel therapeutic strategy for treating hepcidin deficiency-related disorders, including haemochromatosis, particularly in male patients (Yang et al. 2020). Research has shown that cardiac Ca^2+^ and iron levels can be attenuated by an anti-ferroptotic iron chelator, TTCC blocker, deferiprone and efonidipine, and that left ventricular functions are improved in iron-overloaded thalassaemic mice fed a high-iron diet (Khamseekaew et al. 2018). Our study demonstrated that excess systemic haem in SCD patients upregulates Hmox1, which promotes cardiac ferroptosis (Menon et al. 2022). Interestingly, DFP combined with NAC had synergistic therapeutic benefits and exerted more robust beneficial effects than did monotherapy on the basis of its cardioprotective effects. This was shown via restoration of cardiac iron concentration, oxidative stress, and cardiac mitochondrial function, which led to restoration of the cardiac sympathovagal balance, cardiac homeostasis, myocardial contractility and LV function to normal physiological conditions in iron-overloaded rats (Wongjaikam et al. 2016; Wongjaikam et al. 2017). MRI and access to adequate continuous iron chelation therapy aided by periodic LPI measurements that guide chelation therapy should further reduce the risk of cardiac dysfunction and cardiac-related deaths in patients with transfusion-related iron overload, such as TM (Berdoukas et al. 2015).

### Doxorubicin (DOX)-induced cardiac injury (DIC)(Fig. 8)

Clinical use of doxorubicin, an anthracycline drug used to treat tumours, has been limited due to its cardiotoxicity. The pathophysiology of doxorubicin includes inhibition of DNA/RNA/protein synthesis, increased production of ROS, impaired energy metabolism, mitochondrial dysfunction, cardiomyocytes apoptosis, interstitial fibrosis, autophagy disorders and disorders of intracellular calcium homeostasis (Kong et al. 2022a). At present, many studies have shown that ferroptosis is closely related to DIC. Related studies have shown that FUNDC2 knockout mice can resist the decrease in cardiac function and myocardial fibrosis caused by doxorubicin. Knockdown of FUNDC2 inhibits the changes in the morphology of myocardial mitochondria caused by doxorubicin and occurrence of ferroptosis in vivo and in vitro. FUNDC2 can bind to the GSH transporter SLC25A11 on the inner mitochondrial membrane, causing a decrease in the level of GSH in mitochondria and leading to lipid peroxidation and ferroptosis (Ta et al. 2022). A related study showed that after doxorubicin was injected into mice, expression of GPX4 was downregulated, mitochondrial lipid peroxidation was caused by the DOX-Fe^2+^ complex, and mitochondria-dependent ferroptosis was the main cause of DIC. The ferroptosis inhibitor Fer-1 prevents DOX-induced ferroptosis, especially in mitochondria, thus confirming that mitochondria-dependent ferroptosis plays a key role in DIC and that ferroptosis is the main form of regulating cell death (Tadokoro et al. xxxx). Research has shown that reducing mitochondrial iron levels effectively reverses DIC. DOX increases mitochondrial iron levels, and overexpression of the mitochondrial iron output regulator ABCB8 or treatment with DXZ reduces mitochondrial iron levels and cardiac damage from DOX both in vitro and in vivo. To date, dexprazolidine is the only drug recognized by the U.S. Food and Drug Administration (FDA) for prevention of DIC in cancer patients in clinical practice (Ichikawa et al. 2014).

**Fig. 8:**
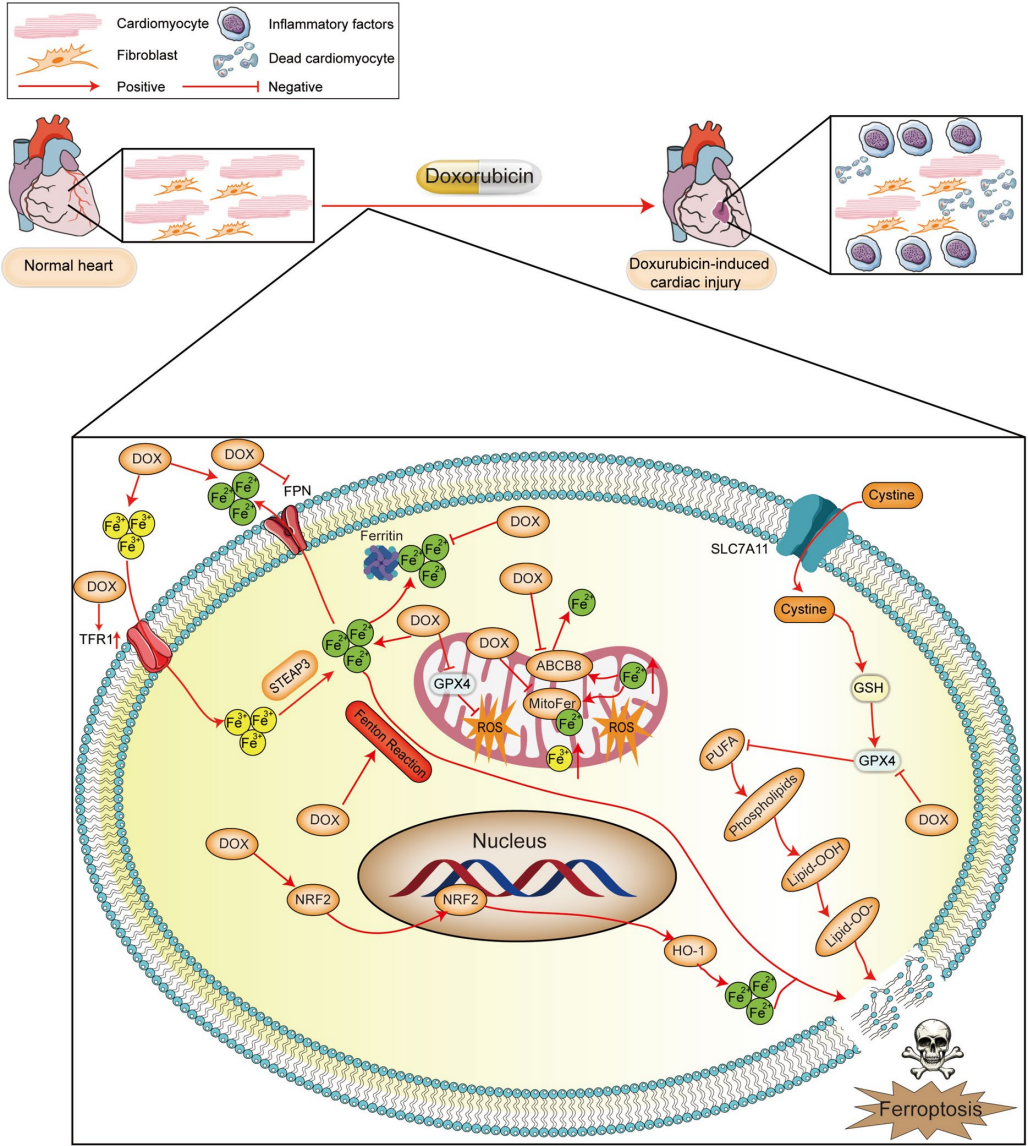
Doxorubicin-induced ferroptosis in cardiomyocytes. SLC7A11: solute carrier family 7 membrane 11, TFR1: Transferrin Receptor 1, GSH: glutathione, GPX4: Glutathione peroxidase 4, PUFA: polyunsaturated fatty acids, ABCB8: ATP-binding cassette protein isoform 8, ROS: reactive oxygen species, NRF2: NF-E2-related factor 2, FPN: Ferroportin, STEAP3: Six-Transmembrane Epithelial Antigen of Prostate 3, HO-1: heme oxygenase 1, DOX: Doxorubicin

### Sepsis-induced cardiac injury

Sepsis is a serious disease that endangers human health. Septic cardiomyopathy is a serious complication, but its pathophysiology can be reversed. Research has shown that ferroptosis is involved in the pathophysiology of septic cardiomyopathy. The four major pathophysiologies of sepsis are endothelial dysfunction, coagulation abnormalities, cellular functional changes, and cardiovascular response disorders (Evans 2018). Related research has shown that ferroptosis is involved in septic cardiomyopathy both in vivo and in vitro and that upregulation of NCOA4 promoted ferritin autophagy and caused ferroptosis but that inhibiting autophagy reduces the occurrence of ferroptosis. Finally, the interaction between NCOA4 and ferritin was confirmed through an immune coprecipitation experiment (Li et al. 2020c). In addition, related research has shown that TMEM43 knockdown exacerbates LPS-induced ferroptosis. Ferrostatin-1 inhibited TMEM43 knockdown-induced exacerbation in vivo. In contrast, an apoptosis inhibitor cannot inhibit the protective effects of TMEM43 (Chen et al. 2022). Knockout of ICA69 ameliorates LPS-induced cardiac damage by suppressing STING-mediated inflammation and ferroptosis in septic cardiomyopathy (Kong et al. 2022b).

### Diabetes-related cardiomyopathy

Diabetes-related cardiomyopathy (DCM) is the main cause of death in diabetic patients. Due to accumulation of cardiac triglycerides, myocardial lipotoxic injury and production of lipid toxic substances such as MDA and 4-HNE lead to ferroptosis. Use of several ferroptosis inhibitors has verified the relationship between ferroptosis and DCM. Related research has shown that vitamin E administration leads to a significant decrease in apoptosis, lipid peroxidation, protein oxidation and the QT interval, which strongly suggests that this free radical scavenger might promote rehabilitation of diabetic cardiomyopathy by reducing oxidative stress and eliminating apoptotic signals, which was confirmed by the restoration of normal QT intervals (Hu et al. 2021b). Related research has shown that CoQ_10_ can reduce diastolic dysfunction, cardiomyocyte hypertrophy, myocardial fibrosis and cell death caused by diabetes. The cardioprotective effect of CoQ_10_ might be mediated through its ability to inhibit systemic oxidative stress, improve cardiac bioenergetics and maintain Akt and SERCA2a regulation in the context of diabetes (Gu et al. 2017).

### Radiation-induced cardiomyopathy

Research has confirmed that ionizing radiation (IR) induces ferroptosis in four ways: 1) inhibiting expression of SLC7A11 in an ATM-dependent manner to trigger ferroptosis by reducing cystine uptake and GSH synthesis, 2) promoting biosynthesis of polyunsaturated fatty acid phospholipids by upregulating expression of ACSL4, 3) inducing lipid peroxidation by producing excessive ROS, and 4) activating autophagy through the cGAS signalling pathway to induce ferroptosis (Lei et al. 2020).

Research has shown that IL-6 and chemokine C–C motif ligand 2 (CCL2) levels increase in human umbilical vein endothelial cells (HUVECs) when the radiation dose is as low as 0.5 Gy, if the radiation dose increases to 2 Gy, levels of IL-8, TGF-β, TNF-α, IL-1β, IFN-γ, and adhesion molecules such as ICAM-1, VCAM-1, E-selectin and VEGF increase significantly (Baselet et al. 2017). Our study proposed a possible new mechanism in the RPE in a laser-induced CNV model, namely, SLC7A11, and its ability to suppress ferroptosis. SLC7A11 may play an antioxidant role, protect cells from ferroptosis, and reduce CNV incidence by activating or increasing GPX4. This provides a new therapeutic approach for neovascular AMD patients who are clinically insensitive to anti-VEGF treatment or who require repeated injections that cause side effects (Zhao et al. 2021).

Research has confirmed that the cGAS-STING pathway is activated after 2 Gy radiation treatment in human coronary artery endothelial cells (HCECest2) (Philipp et al. 2020). Radiation-induced myocardial fibrosis can be diagnosed more than 10 years after radiation therapy even if the patient’s symptoms are often asymptomatic (Heidenreich et al. 2003). In animal models, several months are needed for individuals to develop myocardial fibrosis after irradiation (Darby et al. 2010).

### Heart transplantation

With the increase in the number of heart transplants worldwide, the therapeutic outcomes of patients with advanced heart failure have significantly improved. However, new problems have arisen: I/R injury (IRI) after heart transplantation is a significant type of aseptic inflammation, and the primary graft dysfunction mediated by IRI causes complications in and even kills up to 28% of heart transplant recipients (Kobashigawa et al. 2014). At present, some studies suggest that use of ferroptosis inhibitors might reduce IRI and improve the prognosis of IRI after heart transplantation. Related research has shown that Fer-1 can improve prognosis after cardiac transplantation, and the authors confirmed that the mechanism is not related to necrosis. Inhibiting ferroptosis had the same effect on myocardial ischaemia‒reperfusion mice after coronary artery ligation. Live imaging technology and subsequent experiments confirmed that inhibiting ferroptosis or targeting the TLR4/Trif/type I IFN pathway might be feasible clinical strategies for improving the prognosis of cardiac transplant recipients and patients with ischaemic myocardial reperfusion after coronary artery occlusion (Li et al. 2019b). A multicentre clinical trial of all 103,299 paediatric and adult heart transplants between 1982 and June 2011 revealed that cardiac allograft vasculopathy (CAV) was the main cause of graft failure and death. The incidence of CAV is as high as 20%-65% (Lund et al. 2013). Endothelial dysfunction caused by allograft injury is the main cause of CAV. Ferroptosis is involved in endothelial cell dysfunction, thereby damaging and affecting all parts of the cardiac vascular tree and leading to progression of intima and plaques (Bai et al. 2020; Mallah et al. 2020). Therefore, ferroptosis inhibitors can be used to reduce inflammation during myocardial transplant-related surgery.

### Atrial fibrillation

Atrial fibrillation (AF) is the most common arrhythmia worldwide and affects 1% of the population (Reddy et al. 2022). Mechanical function and electrical activity progressively deteriorate in iron-overloaded hearts. Iron overload can affect calcium, sodium, and potassium channels, interfering with cardiac electrophysiology and confirming the connection between iron ions and arrhythmia (Siri-Angkul et al. 2018). Iron overload caused cardiac mitochondrial dysfunction, as indicated by increased ROS production, mitochondrial membrane depolarization and mitochondrial swelling, and only the mitochondrial calcium uniporter completely protects against the cardiac mitochondrial dysfunction caused by iron overload. The combination of the two causes AF (Sripetchwandee et al. 2014). Previous studies have shown that rats with chronic iron overload develop heart blockage, longer progesterone receptor (PR) intervals, and AF (Rose et al. 2011). Frequent excessive drinking can activate ferroptosis and increase the induction rate of AF. However, the ferroptosis inhibitor Fer-1 can ameliorate iron overload disorders, reduce production of ROS, and ultimately reduce susceptibility to AF (Dai et al. 2022). Sepsis is also one of the causes of AF. Relevant research has shown that Fpn-mediated ferroptosis is involved in new-onset AF with LPS-induced endotoxaemia. Targeting Fpn or inhibiting ferroptosis may be promising treatment strategies for new-onset AF induced by sepsis in the future (Fang et al. 2021). Our results suggest that CF-exos-miR-23a-3p may promote ferroptosis. Moreover, development of persistent AF may be prevented by intervention with exosomal miRNAs to reduce oxidative stress injury and ferroptosis according to bioinformatics analysis and experimental verification (Liu et al. 2022a). Despite limited information regarding the occurrence of arrhythmias with iron overload before development of HF, a study has demonstrated that arrhythmia significantly increases as myocardial iron is deposited in patients with β-thalassaemia and that left ventricular systolic function is preserved, which suggested the independent arrhythmogenic effect of iron toxicity to some extent (Lu et al. 2013).

Increased iron stores, independent of haemochromatosis genotype and inflammation, are associated with prolongation of the QTc interval in men. This is a novel finding. In addition, a meta-analysis showed a prolonged QT interval in thalassaemia major patients compared to healthy controls (Henriksen et al. 2016).

Viral RNA and spike protein can be detected in SAN cells in the hearts of infected hamsters. We established an efficient strategy to derive functional human SAN-like pacemaker cells from hESCs, which express pacemaker markers and display SAN-like action potentials. Furthermore, SARS-CoV-2 infection causes dysfunction of human SAN-like pacemaker cells and induces ferroptosis. Two drug candidates, deferoxamine and imatinib, were identified from a high-content screen as being able to block SARS-CoV-2 infection and infection-associated ferroptosis (Han et al. 2022).

### Pulmonary arterial hypertension

Pulmonary hypertension (PH) is a group of conditions that lead to right ventricular failure and premature death due to elevated pulmonary artery pressure and increased pulmonary vascular resistance. Loss of the pulmonary vascular bed and obstructive remodelling are the causes of elevated pulmonary artery pressure and total peripheral resistance (PVR), leading to progressive right heart failure and decreased function (Humbert et al. 2019).

Iron deficiency has been reported in 43–63% of patients with IPAH and is associated with reduced exercise capacity and increased mortality, suggesting that dysregulated iron metabolism may play an unrecognized role in influencing development of IPAH (Rhodes et al. 2011). A comparative omics and bioinformatics analysis of lung tissue from PAH patients and normal individuals revealed 7 ferroptosis-related genes, indicating that the pathological and physiological processes of PAH involve ferroptosis (Zhang and Liu 2021). PAH pathogenesis involves three main pathophysiologies: pulmonary endothelial cell (EC) dysfunction, pulmonary artery smooth muscle cell (PASMC) proliferation, and right ventricular hypertrophy.

Research has shown that pulmonary artery endothelial ferroptosis triggers NLRP3 inflammasome activation and the initial inflammatory response via the HMGB1/TLR4 pathway in MCT-treated rats. Based on these findings, treating PH with a ferroptosis inhibitor (such as Fer-1) or exploring new medicines based on ferroptosis regulation might inactivate the NLRP3 inflammasome and prevent release of inflammatory factors, thus attenuating progression of PH. This strategy might be promising for treating PH in the future (Xie et al. 2022). SLC7A11 is upregulated in Sugen 5416/hypoxia-induced PAH rats and patients with PAH. Moreover, SLC7A11 inhibits ferroptosis and promotes proliferation by overexpressing SLC7A11 in PASMCs. Additionally, ubiquitin aldehyde binding 1 (OTUB1), the main regulator of SLC7A11 stability, was found to be involved in PASMC ferroptosis and proliferation. Furthermore, erastin induces ferroptosis by inhibiting SLC7A11 and GPX4 expression in vivo and in vitro, suggesting that continuous proliferation of hypoxic PASMCs might be reversed by erastin (Hu et al. 2022). Metabolomic analysis of the right ventricular myocardium in MCT-induced PH rats revealed that ferroptosis is associated with decompensated right ventricular hypertrophy, and dysregulation of iron homeostasis, GSH metabolism, and lipid peroxidation may lead to right ventricular decompensation (Veerdonk et al. 2016). NLRP3-macrophage activation occurs in decompensated RVs in preclinical PAH models and patients with PAH. Inhibiting GP130 or NLRP3 signalling improves RV function. HMGB1 released by PAECs after ferroptosis can activate the NLRP3 inflammasome (Al-Qazazi et al. 2022).

### Heart failure

Heart failure (HF) progresses to the final stage of various CVDs, and the contractile or diastolic force of the heart is weakened. Due to irreversible loss of terminally differentiated cardiomyocytes in HF, early prevention of cardiomyocyte hypertrophy and death is expected to maintain heart function and delay HF (Tsao et al. 2023). Iron deficiency or overload can disrupt the iron homeostasis of cardiomyocytes, leading to HF, and cardiomyocytes are highly susceptible to the influence of free iron overload. Ferroptosis pathways can also regulate the pathophysiology of HF (such as inflammation and cardiac toxicity damage) (Fang et al. 2023).

Fth-deficient cardiomyocytes exhibit reduced expression of the ferroptosis regulator SLC7A11, and overexpressing SLC7A11 selectively in cardiomyocytes increases GSH levels and prevents cardiac ferroptosis (Fang et al. 2020). CircSnx12 can act as an endogenous sponge to bind with miR-224-5p, and the 3'UTR of FTH1 also contains miRNA binding sites. A circRNA-miRNA‒mRNA regulatory network was successfully constructed by identifying differentially expressed genes (DEGs) related to iron metabolism. This new approach revealed potential circRNA targets for treatment of HF (Zheng et al. 2021a). Furthermore, the results of integrated bioinformatics analysis revealed that TLR4 and NADPH oxidase 4 (NOX4) are among upregulated DEGs, and their interaction was inferred from the DEG-associated protein‒protein interaction (PPI) network. either TLR4 or NOX4 knockdown significantly improved left ventricular remodelling and reduced myocyte death. Simultaneously, activated autophagy and ferroptosis in rats with HF were markedly inhibited by either TLR4 or NOX4 knockdown, suggesting that TLR4-NOX4 is a potential therapeutic target for HF through the inhibition of autophagy- and ferroptosis-mediated cell death (Chen et al. 2019). MiR-375-3p is an important factor that induces myocardial fibrosis after MI and accelerates ferroptosis in cardiomyocytes and promotes fibrosis by downregulating GPX4, and this process can be reversed by a miR-375-3p inhibitor or ferroptosis inhibitors. Therefore, intervention via the miR-375-3p/GPX4 signalling pathway can alleviate IR-induced CF by reducing ferroptosis in cardiomyocytes (Zhuang et al. 2022).

## Treatment (Fig. 9)

### Appropriate low-iron diet and changing lifestyle habits

Under normal physiological conditions, body iron concentrations range from 3–5 g. Deviations from this range can lead to either iron deficiency or iron overload and can have pathological consequences (Anderson and Frazer 2017). Population-based studies have examined the hypothetical association between dietary iron intake and heart disease risk (Table 3), but inconsistent results have been found. Nevertheless, a meta-analysis of these prospective cohort studies suggested that higher dietary intake of haem iron is associated with a greater risk of CVDs mortality. Reducing consumption of haem iron may help to prevent premature death due to CVDs (Fang et al. 2015; Han et al. 2020b). Therefore, appropriate low-iron diets may help to prevent CVDs caused by ferroptosis.

**Fig. 9:**
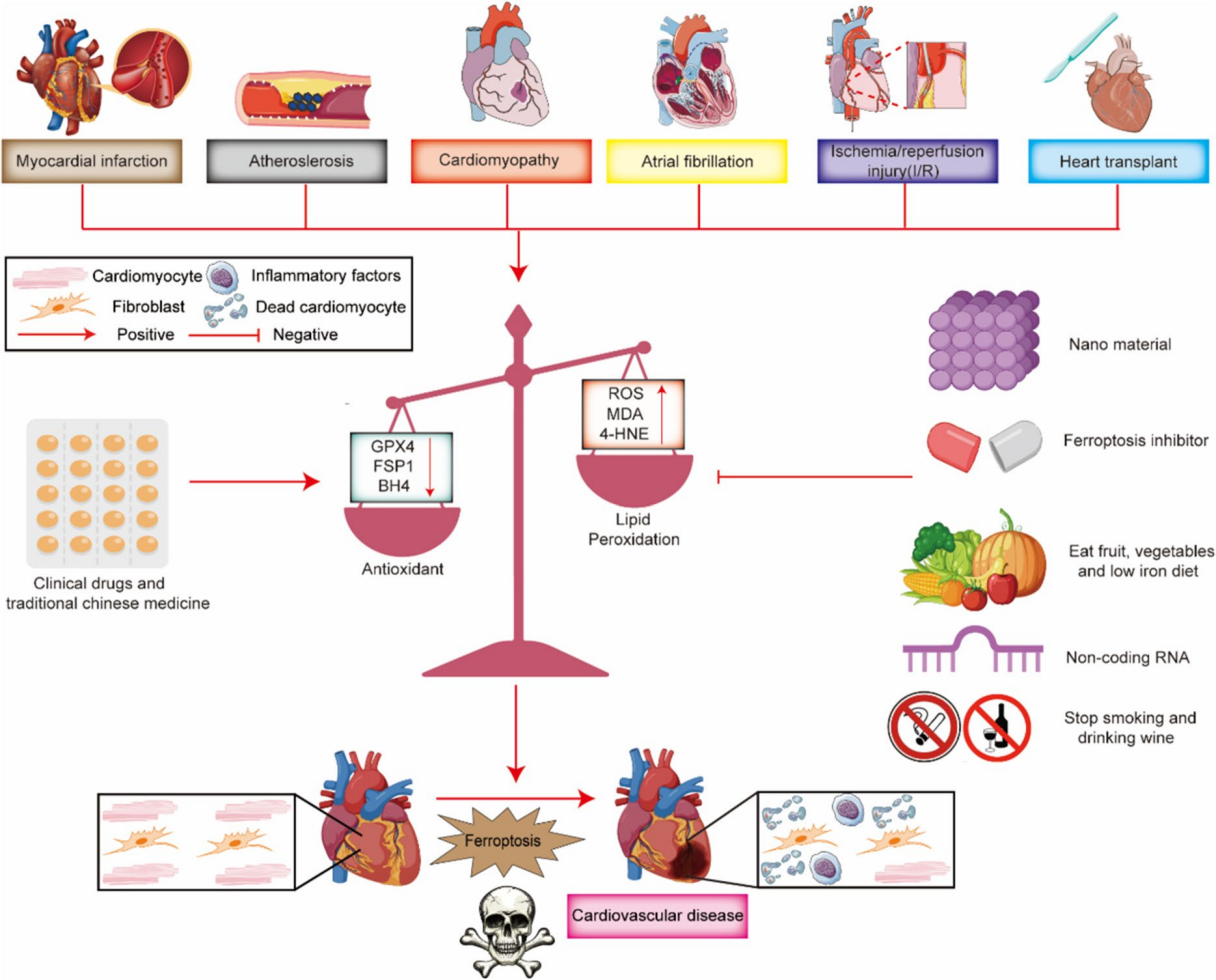
Treatment efficacy and pathological mechanism involved in ferroptosis and CVD. GPX4: glutathione peroxidase 4, FSP1: ferroptosis suppressor protein 1, BH4: tetrahydrobiopterin, ROS: reactive oxygen species, MDA: malonaldehyde, 4-HNE: 4-hydroxynonenal

**Table 3:** Prospective cohort studies of dietary iron intake in CVDs patients

Study	Year	Location	Number of patients	Age(years)	Sex	Follow-up	Disease setting	Findings	References
HPFS	1994	USA	44,933	40–75	Male	4	MI	Dietary iron increases coronary risk in men, they are consistent, however, with an increased risk of myocardial infarction among men with higher intake of haem iron, which is itself positively associated with iron stores	(Ascherio et al. 1994)
NHANES-I	1994	USA	4,237	40–74	Both	13	CHD	These findings from a large national sample do not support the hypothesis of a positive iron-coronary heart disease relationship	(Liao et al. 1994)
Athens	1998	Greece	355	-	Both	2	CVDs	The study provides some support for the hypothesis that dietary iron increases the risk of coronary disease and indicates that the causal link may be stronger among older men and particularly older women	(Tzonou et al. 1998)
Rotterdam Study	1999	Netherlands	4,802	> 55	Both	4	MI	A high dietary haem iron intake was related to an increased risk of myocardial infarction, and it may specifically affect the rate of fatality from myocardial infarction	(Klipstein-Grobusch et al. 1999)
NS	2002	Canada	2,198	18–74	Both	8	MI	There was no increased risk for myocardial infarction with high intake of iron and haem iron	(Malaviarachchi et al. 2002)
IWHS	2005	USA	34,492	55–69	Female	15	CVDs	A higher intake of haem iron might be harmful to CVD mortality	(Lee et al. 2005)
Prospect-EPIC	2005	Netherlands	16,136	49–70	Female	4.3	CHD	Middle-aged women with a relatively high haem iron intake had an increased risk of CHD	(van der et al. 2005)
NHS	2007	USA	6,161	30–55	Female	8.8	CHD	Higher consumption of haem iron and red meat may increase CHD risk among women with type 2 diabetes	(Qi et al. 2007)
LEOGRA	2011	Italy	906	44–79	Female	10	CVDs	Low iron intake seems to be an independent predictor of cerebrovascular and coronary morbidity and mortality in women	(Casiglia et al. 2011)
MESA	2012	USA	5,285	45–84	Both	6.2	CVDs	Positive associations of dietary Zn and haem iron, only from red meat sources, with risk of MetS and CVDs	(Oliveira Otto et al. 2012)
JACC	2012	Japan	58,615	40–79	Both	14.7	CVDs	The findings lend support to the hypothesis that excess iron intake increases CVDs risk	(Zhang et al. 2012)
COSM	2014	Sweden	36,882	45–79	Male	11.7	MI	A high haem iron intake, particularly with simultaneous low intake of minerals that can decrease iron absorption, may increase the risk of fatal AMI	(Kaluza et al. 2014)
The Jiangsu Nutrition Study	2017	China	8,291	20–95	Both	9	CVDs	Both low and high intake of Fe as percentage of RNI were associated with increased risk of mortality in women. Both Fe intake and ferritin levels were not associated with mortality in men	(Shi et al. 2017)
NHANES	2021	USA	14,826	> 18	Both	9.3	CVDs	Interactive effect of holistic dietary vitamins and iron played a protective role in decreasing all-cause and CVDs mortality	(Wang et al. 2021a)
CHNS	2022	China	12,245	> 18	Both	18	Hypertension	Maintaining an appropriate intake level of dietary iron for primary prevention of hypertension	(Zhang et al. 2022c)
CHNS	2023	China	17,107	18–80	Both	26	CVDs	Moderate dietary iron intake may protect against nonfatal CVDs and stroke, especially in Chinese men consuming plant-based diets	(Chen et al. 2023)
CHAMP	2023	Australia	1,705	> 75	Male		CVDs	Higher haem iron intake was independently associated with increased risks of major adverse cardiovascular events	(Luong et al. 2023)

*HPFS* health professionals follow-up study, *NHANES-I* the first national health and nutrition examination survey, *NS* nova scotian, *IWHS* iowa women’s health study, *Prospect-EPIC* european prospective investigation into cancer and nutrition, *CHD* coronary heart disease, *NHS* nurses’ health study, *LEOGRA* the last evidence of genetic risk factors in the aged, *MESA* the multi-ethnic study of atherosclerosis, *MetS* metabolic syndrome, *JACC* the japan collaborative cohort, *COSM* cohort of Swedish men, *AMI* acute myocardial infarction, *NHANES* national health and nutrition examination survey, *CHNS* the China health and nutrition survey, *CHAMP* the concord health and ageing in men project, *CVD* cardiovascular diseases, *MI* myocardial infarction

Frequent excessive alcohol consumption is associated with increased susceptibility to AF compared with single excessive alcohol consumption. Ferroptosis is involved in the initiation of AF induced by excessive alcohol consumption, and inhibition of ferroptosis can reduce susceptibility to AF under these conditions (Dai et al. 2022). In addition, Beclin1 haploinsufficiency enhances acute ethanol challenge-induced myocardial remodelling and contractile dysfunction in a ferroptosis-mediated manner (Liu et al. 2022b). As mentioned above, CSE-induced cell death in rat VSMCs can be completely inhibited by specific ferroptosis inhibitors and an iron chelator. Moreover, CSE induces upregulation of PTGS2 mRNA expression, lipid peroxidation, and intracellular GSH depletion, which are key features of ferroptosis (Sampilvanjil et al. 2020). Cigarette tar promotes atherosclerosis progression by inducing macrophage ferroptosis via the NF-κB-activated hepcidin/FPN/SLC7A11 pathway (Bao et al. 2023). Many ferroptosis inhibitors (cyanidin-3-glucoside, baicalin, vitamin E, etc.) are found in a wide variety of vegetables and fruits (Fan et al. 2021a; Shan et al. 2021; Violi et al. 2022). Therefore, changing unhealthy behaviours (such as quitting smoking and drinking) and eating vegetables and fruits may help to prevent the CVDs caused by ferroptosis.

### Clinical drugs and traditional Chinese medicine

(Table 4)

**Table 4:** Clinical drugs and traditional Chinese medicine treatment as a target for CVDs involving ferroptosis

Disease	Factors	Mechanisms	Role	References
Ischaemia/reperfusion (I/R) injury	Cyanidin-3-glucoside	Decreases Fe^2+^, downregulates TFR1 expression, upregulates Fth1 and GPX4 expression	Inhibits ferroptosis and reduces myocardial injury	(Shan et al. 2021)
	Naringenin	Adjusts the NRF2/system xc-/GPX4 axis	Inhibits ferroptosis and reduces myocardial injury	(Xu et al. 2021)
	Dexmedetomidine	Increases expression levels of Nrf2, SLC7A11, and GPX4 Targets the Nrf2/AMPK/GSK-3β signalling pathway	Inhibits ferroptosis and reduces myocardial injury	(Wang et al. 2022b)
	Etomidate	Induces Nrf2 nuclear translocation	Inhibits ferroptosis and reduces myocardial injury	(Lv et al. 2021)
	Propofol	Targets the AKT/p53 signalling pathway	Inhibits ferroptosis and reduces myocardial injury	(Li et al. 2022b)
	Metformin	Targets the AMPK/ERK signalling pathway	Inhibits ferroptosis and reduces myocardial injury	(Wu et al. 2023)
	Resveratrol	Decreases Fe^2+^, downregulates TFR1 expression, upregulates Fth1 and GPX4 expression	Inhibits ferroptosis and reduces myocardial injury	(Li et al. 2022c)
	Shenmai injection	Activates the Nrf2/GPX4 signalling pathway	Inhibits ferroptosis and reduces myocardial injury	(Mei et al. 2022)
	Salvianolic acid B	Activates the Nrf2 signalling pathway	Inhibits ferroptosis and reduces myocardial injury	(Shen et al. 2022)
	Puerarin	Reduces expression of PTGS2, increases GPX4 expression	Inhibits ferroptosis and reduces myocardial injury	(Ding et al. 2023)
	Baicalin	ACSL4-controlled ferroptosis	Inhibits ferroptosis and reduces myocardial injury	(Fan et al. 2021a)
	Honokiol	Activates the SIRT1-Nrf2 signalling pathway	Inhibits ferroptosis and reduces myocardial injury	(Zhang et al. 2018)
	Gossypol Acetic Acid	Increases GPX4 protein levels	Inhibits ferroptosis and reduces myocardial injury	(Lin et al. 2021)
	Geniposide	Activates the NRF2/HO-1 signalling pathway	Inhibits ferroptosis and reduces myocardial injury	(Wang et al. 2019)
	Luteolin	Upregulates Nrf2 and the Nrf2-related antioxidative signalling pathway	Inhibits ferroptosis and reduces myocardial injury	(Xiao et al. 2019b)
	Butin	Activates Nrf2-regulated antioxidant enzymes	Inhibits ferroptosis and reduces myocardial injury	(Duan et al. 2017)
	Ferulic Acid	Upregulates AMPKα2 expression-mediated ferroptosis depression	Inhibits ferroptosis and reduces myocardial injury	(Liu et al. 2021c)
Atherosclerosis	Qing-Xin-Jie-Yu Granule	Targets the GPX4/xCT signalling pathway	Inhibits ferroptosis	(Zhang et al. 2023)
	Tongxinluo	Targets the GPX4/ACSL4/FSP1 signalling pathway	Inhibits ferroptosis	(Wang et al. 2022c)
Hypertension	Monocrotaline	Activates the HMGB1/TLR4/NLRP3 inflammatory pathway	Promotes ferroptosis	(Xie et al. 2022)
	Celastrol	Increases HO-1 expression and decreases ROS production	Reduces inflammation and oxidative stress in VSMCs caused by hypertension	(Yu et al. 2010)
	Elabela	Regulates the IL-6/STAT3/GPX4 signalling pathway	Inhibits AngII-induced ferroptosis in poor myocardial remodelling, fibrosis and cardiac dysfunction	(Zhang et al. 2022d)
	Dihydroartemisinin	Increases IRF3-SLC7A11, decreases ALOX12 and iron levels	Inhibit ferroptosis	(Shi et al. 2022a)
Heart failure	Puerarin	Regulates Nox4 signalling	Inhibits ferroptosis	(Liu et al. 2018)
	Imeglimin	Restores expression of GPX4	Inhibits ferroptosis	(Kitakata et al. 2021)
	Atorvastatin	Inhibits ferritinophagy-mediated ferroptosis	Inhibits ferroptosis	(Ning et al. 2021)
Diabetes cardiomyopathy	Troglitazone	Promotes GPX4 ubiquitination	Resists cell damage caused by high glucose	(Zhang et al. 2021b)
	Canagliflozin	Promotes the system Xc-/GSH/GPX4 axis	Inhibits ferroptosis	(Du et al. 2022)
	Sulforaphane	Targets AMPK/NRF2	Inhibits ferroptosis	(Wang et al. 2022d)
	Curcumin	Targets NRF2/GPX4/HMOX1	Inhibits ferroptosis	(Wei et al. 2022)
	Palmitic acid	Reduces HSF1 and GPX4	Promotes ferroptosis and enhances endoplasmic reticulum stress	(Wang et al. 2021b)
Doxorubicin cardiomyopathy	Empagliflozin	Participates in NLRP3- and MYD88-related pathways	Inhibits ferroptosis, fibrosis, apoptosis and inflammation	(Quagliariello et al. 2021)
	LCZ696	Activates the AKT/SIRT3/SOD2 signalling pathway	Inhibits ferroptosis	(Liu et al. 2022c)
	Melatonin	Regulates YAP expression	Inhibits ferroptosis	(Sun et al. 2022)
	Salidroside	Activates AMPK-dependent signalling pathways	Inhibits ferroptosis	(Chen et al. 2022)
	Fluvastatin	Targets HMG-CoA reductase	Inhibits ferroptosis	(Riad et al. 2009)
	Xanthohumol	Regulates NRF2 and GPX4 protein levels	Inhibits ferroptosis	(Ichikawa et al. 2014)
	Astragaloside IV	Activates Nrf2 signalling pathway and increases GPX4 expression	Inhibits ferroptosis and improves fibrosis	(Luo et al. 2021b)
Sepsis-induced cardiac injury	Puerarin	Targets AMPK-mediated ferroptosis signalling	Inhibits ferroptosis	(Zhou et al. 2022)
	Dexmedetomidine	Reduces HO-1 expression, increases GPX4 expression	Reduces sepsis-induced myocardial cell damage	(Wang et al. 2020b)
Atrial fibrillation	Icariin	Targets SIRT1 signalling	Inhibits ferroptosis	(Yu et al. 2023)

*TFR1* transferrin receptor 1, *Fth1* ferritin heavy chain, *GPX4* glutathione peroxidase 4, *NRF2* nuclear factor erythroid 2-related factor 2, *SLC7A11(xCT)* solute carrier family 7 member 11, *AMPK* amp-activated protein kinase, *GSK-3β* glycogen synthase kinase-3β, *AKT* protein kinase B, *FSP1* ferroptosis-suppressor protein 1, *ROS* reactive oxygen species, *HO-1* haem oxygenase-1, *SIRT1* sirtuin 1, *SIRT3* sirtuin 3, *HMG-CoA* 3-hydroxy-3-methylglutaryl-coenzyme A, *YAP* yes-associated protein, *SOD2* superoxide dismutase 2, *NLRP3* nod-like receptor family pyrin domain containing 3, *MYD88* myeloid differentiation primary response 88, *HSF1* hsf1, *GSH* glutathione, *Nox4* nadph oxidase 4 protein, *ALOX12* arachidonate 12-lipoxygenase, *IRF3* interferon regulatory factor 3, *IL-6* interleukin 6, *STAT3* signal transducer and activator of transcription 3, *HMGB1* high-mobility group box-1 protein, *TLR4* toll-like receptor 4, *ACSL4* acyl-CoA synthetase long-chain family member 4, *AMPKα2* amp-activated protein kinase alpha2, *VSMCs* vascular smooth muscle cells, *ERK* extracellular signal-regulated kinase, *PTGS2* prostaglandin-endoperoxide synthase 2

### Ferroptosis Inhibitors

(Table 5)

**Table 5:** Ferroptosis inhibitor treatment as a target for CVDs involving ferroptosis

Ferroptosis inhibitor	Mechanism	Targets	References
Ferrostatin-1	Eliminates ROS, inhibits lipid peroxidation, and regulates expression of oxidation-related proteins; reduces unstable iron in cells	Doxorubicin-induced cardiomyopathy, myocardial ischaemia‒reperfusion injury, TAC-induced cardiomyopathy, heart transplantation, atherosclerosis, sepsis‑induced cardiomyopathy, palmitic acid-induced myocardial injury, atrial fibrillation, pulmonary arterial hypertension	(Dixon et al. 2012; (Bai et al. 2020; Fang et al. 2019; Li et al. 2020c; Li et al. 2019b; Dai et al. 2022; Wang et al. 2021b; Li et al. 2020d; Song et al. 2024)
Liproxstatin-1	Clears ROS, inhibits lipid peroxidation, activates the Nrf2 signalling pathway, down regulates VDAC1, and restores the GPX4 level	Myocardial Ischaemia‒reperfusion injury, palmitic acid-induced myocardial injury, diabetic cardiomyopathy, sepsis‑induced cardiomyopathy	(Basit et al. 2017; Chen et al. 2022; Wang et al. 2022d; Wang et al. 2021b; Feng et al. 2019)
α-Tocopherol analogues	Clears ROS and inhibits lipid peroxidation	Heart transplantation	(Viswanathan et al. 2017; Kucharska et al. 1998)
Nitrogen oxide	Blocks the Fenton reaction and inhibits production of hydroxyl radical	Diabetic cardiomyopathy	(Friedmann Angeli et al. 2014; Fecht et al. 2022)
Zileuton	Inhibits lipid peroxidation by inhibiting Alox5	Ischaemia‒reperfusion injury	(Yuk et al. 2021; Gonca 2013)
Troglitazone	Specific inhibition of ACSL4	Diabetic cardiomyopathy	(Dachert et al. 2020; Nakajima et al. 1999)
Gastrodin	Activates Nrf2 signal path	Myocardial ischaemia‒reperfusion injury, heart failure	(Griesser et al. 2018; Han et al. 2019; Shu et al. 2012; Zheng et al. 2017; Kang et al. 2021)
Curcumin	Chelates iron, reduces iron accumulation, and activates the Nrf2 signalling pathway	Diabetic cardiomyopathy, myocardial ischaemia‒reperfusion injury	(Yuan et al. 2016; Ren et al. 2020)
Ferroportin	Iron export protein	Sepsis‑induced cardiomyopathy	(Fang et al. 2021; Jiang et al. 2020)
Mito TEMPO	Suppresses lipid peroxidation	Doxorubicin-induced cardiomyopathy, Diabetes cardiomyopathy	(Fang et al. 2019; Ni et al. 2016)
Deferoxamine	Reduces intracellular iron levels and oxidative damage	Myocardial ischaemia‒reperfusion injury, diabetic cardiomyopathy,	(Kose et al. 2019; Abdul et al. 2021; Tu et al. 2021)
Deferiprone	Iron chelator	Sepsis‑induced cardiomyopathy	(Kenny et al. 2019)
Pioglitazone	Specific inhibition of ACSL4	Doxorubicin-induced cardiomyopathy, diabetic cardiomyopathy, heart failure	(Dachert et al. 2020; Pakravan et al. 2022; Gbr et al. 2021; Legchenko et al. 2018)
Rosiglitazone	Specific inhibition of ACSL4	Doxorubicin-induced cardiomyopathy	(Dachert et al. 2020; Saraogi et al. 2010)
Fluvastatin	Targets HMG-CoA reductase	Doxorubicin-induced cardiomyopathy	(Bao et al. 2021; Kuscu et al. 2023)
Lovastatin	Targets HMG-CoA reductase	Doxorubicin-induced cardiomyopathy	(Guo et al. 2022; Feleszko et al. 2000)
Simvastatin	Targets HMG-CoA reductase	Doxorubicin-induced cardiomyopathy	(Rayatpour et al. 2022; Pecoraro et al. 2023)
Dexrazoxane	Inhibits iron overload	Doxorubicin-induced cardiomyopathy, myocardial ischaemia‒reperfusion injury, sepsis‑induced cardiomyopathy	(Fang et al. 2019; Li et al. 2020c)
Salvianolic acid B	Activates NRF2	Myocardial infarction	(Shen et al. 2022)
Britanin	Upregulates GPX4	Myocardial ischaemia/reperfusion injury	(Lu et al. 2022)
Vitamin C	Clears ROS	Myocardial ischaemia/reperfusion injury	(Davis et al. 2016)
Vitamin E	Clears ROS	Myocardial ischaemia/reperfusion injury	(Saleh and Saleh 2010)
Naringenin	Regulates NRF2	Myocardial ischaemia/reperfusion injury	(Zhou et al. 2022)
Dexmedetomidine	Activates NRF2 through the AMPK/GSK-3β pathway	Myocardial ischaemia/reperfusion injury	(Wang et al. 2022b)
Histochrome	Reduces cytosolic and mitochondrial ROS, maintains intracellular GSH levels, and elevates GPX4 activity	Myocardial ischaemia/reperfusion injury	(Dong et al. 2020; Jiang et al. 2021; Ngo and Duennwald 2022)
Melatonin	Regulates YAP	Doxorubicin-induced cardiomyopathy	(Sun et al. 2022)
XJB-5–131	Clears ROS	Myocardial ischaemia/reperfusion injury	(Escobales et al. 2014)
JP4-039	Clears ROS	Myocardial ischaemia/reperfusion injury	(Escobales et al. 2014)

*YAP* yes-associated protein, *NRF2* nuclear factor erythroid 2-related factor 2, *ACSL4* acyl-CoA synthetase long-chain family member 4, *HMG-CoA* 3-hydroxy-3-methylglutaryl-coenzyme A, *AMPK* amp-activated protein kinase, *ALOX5* arachidonate 5-lipoxygenase, *GSK-3β* glycogen synthase kinase-3β, *GSH* glutathione, *GPX4* glutathione peroxidase 4, *ROS* reactive oxygen species, *VDAC1* voltage-dependent anion channel 1, *TAC* transverse aortic constriction

### Noncoding RNAs

(Table 6)

**Table 6:** Noncoding RNA treatment as a target for ferroptosis-related CVDs

Disease	NcRNAs	Sample types	Mechanisms	Role	References
Myocardial infarction	miR-23a-3p	Mouse heart tissues/cardiomyocytes	Inhibits DMT1 expression	Inhibits ferroptosis and reduces myocardial injury	(Song et al. 2021)
	miR-15a-5p	Mouse myocardial tissues/cardiomyocytes	Downregulates GPX4 expression	Inhibits ferroptosis and reduces myocardial injury	(Fan et al. 2021b)
	miR-190a-5p	H9c2 cells/HEK-293 T cells	Inhibits GLS2 expression	Inhibits ferroptosis	(Zhou et al. 2021b)
	miR-223-3p	Mouse myocardial tissues/H9c2 cells	Adjusts the threshold of iron ion induction	Inhibits ferroptosis	(Nishizawa et al. 2020)
	lncRNA-UCA1	Mouse myocardial tissues	Inhibits miR-873-5p/XIAP axis	Inhibits ferroptosis	(Sun et al. 2020)
	lncRNA-p21	Mouse myocardial tissues	Inhibits formation of the p300-p53 complex	Promotes ferroptosis	(Wu et al. 2014)
	lncRNA Gm47283	Mouse heart tissues/HL-1 cells	Targets miR-706/PTGS2/ferroptosis axis	Inhibits ferroptosis	(Gao et al. 2022)
	circRNA1615/miR-152-3p	Mouse heart tissues/HL-1 cells	Promotes LRP6 expression	Inhibits ferroptosis	(Li et al. 2021d)
Reperfusion injury	miR-199a-5p	H9c2 cells	Inhibits the Akt/eNOS signalling pathway	Promotes ferroptosis	(Zhang et al. 2022e)
	lncAABR07025387.1	Mouse heart tissues/HL-1 cells	Targets miR-205/ACSL4-mediated ferroptosis	Promotes ferroptosis	(Sun et al. 2022)
	miR-375-3p	Cardiac fibroblasts	Downregulates GPX4 expression	Promotes ferroptosis	(Zhuang et al. 2022)
	miR-135b-3p	Mouse myocardial tissues	Downregulates GPX4 expression	Promotes ferroptosis	(Sun et al. 2021)
	miR-29b-3p	Mouse myocardial tissues	Inhibits PTX3 pathway	Promotes ferroptosis	(He and Yan 2021)
	miR-30d	Mouse myocardial tissues	Inhibits ATG5 pathway	Promotes ferroptosis	(Tang et al. 2020)
Atherosclerosis	lncRNA-XXYLT1-AS2	HUVECs	targeting the RNA binding protein FUS	Inhibits ferroptosis	(Wang et al. 2020c)
	miR-17–92	HUVECs	Targeting zinc lipoprotein A20 reduces Acsl4 expression and ROS accumulation	Inhibits ferroptosis	(Xiao et al. 2019a)
Heart failure	miR-351	Mouse heart tissues	inhibited the JNK/p53 signalling pathway by targeting MLK3	Inhibits ferroptosis	(Wang et al. 2020d)
	miR-27a/miR-28–3p/miR-34a	Rat myocardial tissues/H9c2 cells	cardiac Nrf2 dysregulation	Inhibits ferroptosis	(Tian et al. 2018)
	lncRNA GAS5/miR-18b-5p/miR-185–5p/miR-29b-3p	Human tissues	GAS5/miR-18b-5p/PLIN2, GAS5/miR-185–5p/LPCAT3, and GAS5/miR-29b-3p/STAT3 were associated with ferroptosis in Heart failure	Inhibits ferroptosis	(Zheng et al. 2021b)
	circSnx12	Mice myocardial tissues/HL-1 cells	Targeting miR-224-5p	Inhibits ferroptosis	(Zheng et al. 2021a)
Diabetes cardiomyopathy	long noncoding RNA ZFAS1	Mouse myocardial tissues	sponging miR-150-5p and activates CCND2	Inhibits ferroptosis	(Ni et al. 2021)
	lncRNA-ZFAS1/miR-150-5p	Mice myocardial tissues/mice primary cardiomyocytes/HEK293T	Downregulated CCND2 expression	Promotes ferroptosis	(Ni et al. 2021)
Doxorubicin cardiomyopathy	lncRNA KCNQ1OT1/miR-7–5p	AC16 cells/rat ventricle cardiomyocytes	Promotes TFR expression	Promotes ferroptosis	(Zhuang et al. 2023)
Sepsis-induced cardiac injury	miR-149	Mice myocardial tissues	Inhibits HMGB1 expression	Inhibits ferroptosis	(Wang et al. 2022e)
Atrial fibrillation	miR-23a-3p	H9c2 cells	Inhibits SLC7A11 expression	Promotes ferroptosis	(Liu et al. 2022a)

*DMT1* divalent metal transporter 1, *GPX4* glutathione peroxidase 4, *GLS2* glutaminase 2, *PTGS2* prostaglandin-endoperoxide synthase 2, *AKT* protein kinase B, *LRP6* lipoprotein receptor-related protein 6, *XIAP* x-linked inhibitor of apoptosis, *eNOS* endothelial nitric oxide synthase, *ACSL4* acyl-CoA synthetase long-chain family member 4, *PTX3* pentraxin 3, *ATG5* autophagy-related gene 5, *FUS* fused in sarcoma, *JNK* c-Jun N-terminal kinase, *MLK3* mixed lineage kinase 3, *HMGB1* high-mobility group box-1 protein, *CCND2* cyclin D2, *LPCAT 3* lysophosphatidylcholine acyltransferase 3, *SLC7A11* solute carrier family 7 member 11, *STAT3* signal transducer and activator of transcription 3, *PLIN2* perilipin 2

## Nanomaterial

Use of nanomaterials as an emerging and innovative therapeutic technology for CVDs treatment. Application of CVDs nanomedicine depends on the expected clinical effects, including 1) combining nanomaterials to improve the function (mechanical, immune, electrical) of the heart or related biomaterials, 2) administering nanotherapy and imaging diagnosis of vascular systems, nanomaterials or tissue nanoengineering solutions, and 3) improving the sensitivity and/or specificity of in vitro diagnostic methods for patient disease diagnosis (Zhong et al. 2022; Shi et al. 2022b; Luo et al. 2021c).

MMPP protects the heart against sepsis-induced myocardial injury by inhibiting ferroptosis and inflammation and might be a novel therapeutic approach in the future (Liu et al. 2023). Ferroptosis is a novel mechanism for ZnONP-induced endothelial cytotoxicity, and NCOA4-mediated ferritinophagy is required for ZnONP-induced ferroptotic cell death (Qin et al. 2021). We developed a delivery system based on a neutrophil membrane (NM)-camouflaged mesoporous silica nanocomplex (MSN) for inhibition of cardiac hypertrophy, indicating the potential role of silencing lncRNA AAB (si-AAB) and overexpressing miR-30b-5p as novel therapies for cardiac hypertrophy (Shi et al. 2022). Myocardial myocardium-targeted nanomedicine significantly protects the heart from I/R injury before irreversible pathological changes occur (Yang et al. 2022).

## Conclusions

In conclusion, many basic and clinical studies have demonstrated the role of ferroptosis in CVDs, and lipid peroxidation, iron accumulation and amino acid metabolism are obvious features of ferroptosis, indicating that ferroptosis is closely related to the pathophysiology of CVDs. Ferroptosis, which is a proinflammatory reaction, links oxidative stress and inflammatory reactions and has led to some progress in CVDs treatment. Moreover, several drugs, such as dexrazoxane, have been developed to reduce CVDs incidence through ferroptosis. However, the mechanism underlying the association between ferroptosis and CVDs incidence is still unclear, and most related work involves basic research. Overall, further elucidation of the mechanism of ferroptosis is needed, as are additional clinical studies to confirm the connection between ferroptosis and CVDs incidence.

## Limitation

At present, research on ferroptosis has been mostly based on basic research such as cell and animal experiments, and there has been no relevant clinical evidence based onmedicine. Furthermore, there is no gold standard target for ferroptosis in basic research, which has resulted in many difficulties in transforming basic experiments into clinical and evidence-based medicine. The ferroptosis biomarkers currently used in preclinical studies are nonspecific and function in other types of cell death and certain pathological conditions. When and how do other forms of cell death occur together with ferroptosis in development of CVDs? Moreover, what are the key protective mechanisms to prevent ferroptosis in the heart? On the other hand, there are still many unanswered questions in the basic research of ferroptosis, such as the specific relationship between ferroptosis and autophagy, and whether lipid autophagy regulation participates in ferroptosis. The relationship between ferroptosis and inflammation is unclear, and inflammation and ROS caused by ferroptosis are sometimes not harmful and can even be beneficial. The specific mechanism of ferroptosis needs to be further studied. Finally, there are some ferroptosis inhibitors and inducers that have not been verified in basic experiments in other diseases, and further research should be carried out in CVDs to translate these agents into clinical use. There are still unknown targets, which might bring more possibilities for targeted clinical treatment of CVDs. Is it possible to design effective targeted strategies for ferroptosis to prevent and treat CVDs related to ferroptosis? As described above, ferroptosis is driven by peroxidation of specific PUFA-containing lipids in particular organelles, such as the ER. How, where and when this leads to cell death per se are unknown.

## Future

First, research on ferroptosis is mostly based on basic research such as cell and animal experiments, with little clinical evidence based on clinical experiments and evidence-based medicine. In fact, the most important unresolved issue is how ferroptosis kills cardiomyocytes, and it is not yet clear which cell membranes need to be destroyed to cause ferroptosis. In addition, the in-depth molecular interactions and mechanisms of different heart cells need to be elucidated through in-depth multi omics analysis and functional screening. Second, it is necessary to study the gold standard targets of ferroptosis, which leads to theoretical research related to ferroptosis being able to serve clinical life. In addition, there are still many unanswered questions in the basic research of ferroptosis, such as the specific relationship between ferroptosis and autophagy, and whether lipid autophagy regulation is involved in ferroptosis. The relationship between ferroptosis and inflammation is still unclear, and the inflammation and ROS caused by ferroptosis are sometimes harmless or even beneficial. Ferroptosis appears to be a double-edged sword, and equally important is addressing whether use of ferroptosis disease activators can alleviate inflammation, autophagy, or ROS in certain situations. As a defence response to injury, inflammation is not always harmful, and the specific mechanism of ferroptosis warrants further research. Once again, is it possible to design effective targeted strategies for ferroptosis to prevent and treat CVDs related to iron deficiency? For example, microbubbles, as a novel carrier for genes or drugs, have been widely used in research of CVDs. Based on current research on ferroptosis, we assume that using microbubbles as carriers to carry drugs or genes that block ferroptosis and target them to an area under ultrasound action will greatly improve drug utilization and gene transfection efficiency, which will benefit precise treatment of CVDs. Is there ferroptosis in physiological processes and its significance? Finally, there are some iron- removal inhibitors and inducers that have not been validated in basic experiments of other diseases that should be further studied in CVDs to convert these drugs into clinical applications. Moreover, we will use antioxidants and iron-chelating agents to conduct scientific research and validation in animals simultaneously, seeking an appropriate and novel target dose drug. Although research will elucidate the mechanisms of action of these drugs, as well as the associations and characteristics of various mechanisms, there are still unknown targets, which may bring more possibilities for targeted clinical treatment of CVDs. Additionally, understanding the mechanism of iron metabolism and pro-ferroptotic signal propagation between organelles (ER, Golgi apparatus, lipid droplets, and nucleus) may contribute to novel insights, such as how organelles communicate with each other during ferroptosis and whether the occurrence and transformation of specific organelles affect the susceptibility of cells to ferroptosis. Therefore, elucidating the specific role of organelles in ferroptosis will provide a new direction for treatment of CVDs. 

Cell–cell contacts inhabits ferroptosis by decreasing lipid peroxidation. Is Hippo pathway the only mechanism involved in the high-density-induced resistance to ferroptosis of the adherent cells? How the cell-to-cell interplay can be used to enhance the efficacy of the ferroptosis inducers? Does E-cadherin expression also trigger ferroptosis resistance in other cell clusters? Additionally, how is the signal integrated in clustered cells expressing both E- and N-cadherin? 

Cells secrete factors that strongly activate the innate immune system through ferroptosis, playing a role in regulating cell inflammation, signal transduction, and cell growth. When the inflammatory response exceeds a certain limit, a large amount of pro-inflammatory cytokines are released, which will harm human health. At present, corresponding drugs such as ferroptosis inhibitors, antioxidants, and iron chelators have been developed in basic experiments to exert anti-inflammatory and inhibitory effects on ROS generation. Given the close relationship between ferroptosis and inflammation, a multi-mechanism treatment combining anti-inflammatory and anti-ferroptosis may achieve a therapeutic effect of 1+1>2.

Firstly, as the specific mechanisms by which ferroptosis is involved in various. inflammatory diseases have not been fully elucidated, it is difficult to determine whether the effects of ferroptosis inhibitors or drugs targeting ferroptosis are specific to specific categories of CVDs with unique characteristics or are typically applicable to most CVDs. Therefore, a deeper understanding of the mechanisms related to ferroptosis and inflammation will help achieve this goal. For example, what is the specific mechanism of DAMP released by ferroptosis cells? What is the mechanism that leads to immune cell activation? Secondly, ferroptosis is intertwined with other regulatory cell death and phenotypes in CVDs. It is particularly important to develop specific targeted treatments for ferroptosis in CVDs while avoiding systemic adverse reactions.

Finally, due to the extensive clinical use of anti-inflammatory drugs in CVDs, further large-scale and multi-center clinical trials can be conducted to further confirm whether ferroptosis is related to inflammation treatment.

## Data Availability

The datasets generated during and/or analysed during the current study are available from the corresponding author upon reasonable request.

## Abbreviations

ACD: Accidental cell death
RCD: Regulated cell death
ROS: Reactive oxygen species
GSH: Glutathione
GPX4: Glutathione peroxidase 4
PTGS2: Prostaglandin-endoperoxide synthase 2
FSP1: Ferroptosis suppressor protein 1
NRF2: Nuclear factor erythroid 2-related factor2
RIPK1: Recombinant receptor-interacting serine threonine kinase 1
FADD: Fas-associated death domain
TNF-α: Tumour necrosis factor-α
TF: Transferrin
FTH: Ferritin heavy chain
FPN1: Ferritin 1
SLC7A11: Solute carrier family 7 member 11
4-HNE: 4-Hydroxynonenal
PE: Phosphatidylethanolamine
AdA: Arachidonic acid
AdA-CoA: Adrenoyl-CoA
LPCAT3: Recombinant lysophosphatidylcholine acyltransferase 3
LOX: Lipoxygenase
RSL3: Methyl(1S,3R)-2-(2-chloroacetyl)-1-(4-methoxycarbonylphenyl)-1,3,4,9-tetrahydropyrido[3,4-b]-indole-3-carboxylate
PHD1: Hypoxia-inducible factor hydroxylase 1
LAMP2A: Lysosome-associated membrane protein type 2A
HSPA5: Heat shock protein family A member 5
VDAC1: Voltage-dependent anion channel 1
NCOA4: Nuclear receptor coactivator 4
BCL-2: B-cell lymphoma-2
BAX: BCL-2-associated protein X
NAIP: Neuronal apoptosis inhibitory protein
NCCD: The Nomenclature Committee on Cell Death
NLRP3: Nod-like receptor thermal protein domain-associated protein 3
CAMKII: Calcium/calmodulin-dependent protein kinase II
RIPK3: Recombinant receptor-interacting serine threonine kinase 3
MLKL: Mixed lineage kinase domain-like
MPTP: 1-Methyl-4-phenyl-1.2.3.6-tetrahydropyridine
TFR: Transferrin
DMT1: SLC11A2
FTL: Ferritin light chain
GSSG: Glutathione
SLC3A2: Solute carrier family 3 member 2
MDA: Malonaldehyde
PUFAs: Polyunsaturated fatty acids
AA-CoA: Arachidonic acid-CoA
AA: Arachidonic acid
ACSL4: Acyl-CoA synthetase long-chain family member 4
TPD52: Tumour protein D52
ARNTL1: Aryl hydrocarbon receptor nuclear transporter 1
CMA: Molecular chaperone-mediated autophagy
HSPA8: Heat shock protein family A member 8
IFSP1: 1-Amino-3-(4-methylphenyl) pyrido [1,2-a] benzimidazole-2,4-dicarbonitrile
USP7: Ubiquitin-specific protease 7
